# An electrochemical modification strategy to fabricate NiFeCuPt polymetallic carbon matrices on nickel foam as stable electrocatalysts for water splitting†

**DOI:** 10.1039/d2sc02845j

**Published:** 2022-07-05

**Authors:** Ziqi Zhang, Yiduo Li, Zhe Zhang, He Zheng, Yuxin Liu, Yuxing Yan, Chunguang Li, Haiyan Lu, Zhan Shi, Shouhua Feng

**Affiliations:** State Key Laboratory of Inorganic Synthesis and Preparative Chemistry, College of Chemistry, Jilin University Changchun 130012 P. R. China zshi@mail.jlu.edu.cn luhy@jlu.edu.cn

## Abstract

Electrochemical modification is a mild and economical way to prepare electrocatalytic materials with abundant active sites and high atom efficiency. In this work, a stable NiFeCuPt carbon matrix deposited on nickel foam (NFFeCuPt) was fabricated with an extremely low Pt load (∼28 μg cm^−2^) using one-step electrochemical co-deposition modification, and it serves as a bifunctional catalyst for overall water splitting and achieves 100 mA cm^−2^ current density at a low cell voltage of 1.54 V in acidic solution and 1.63 V in alkaline solution, respectively. In addition, a novel electrolyte was developed to stabilize the catalyst under acidic conditions, which provides inspiration for the development of highly efficient, highly stable, and cost-effective ways to synthesize electrocatalysts.

## Abstract 2

Multiple metal elements immobilized into a carbon matrix to fabricate an ultra-stable water splitting electrocatalyst by one-step electrochemical modification.

## Graphical abstract

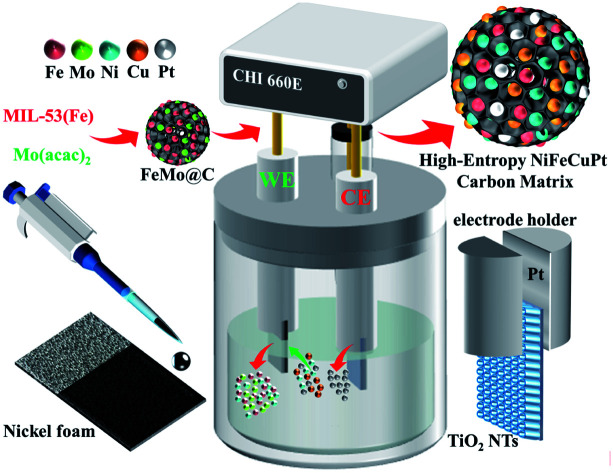

## Introduction

Hydrogen is one of the most desirable alternatives to fossil fuels due to its high energy density, carbon neutrality and environmental friendliness.^1^ Compared with the steam reforming process which accelerates fossil fuel depletion and CO_2_ emissions,^2^ electrochemical water splitting (EWS) is a desirable way to produce highly purified H_2_ since it can be powered by renewable energies without CO_2_ emissions.^3^

To drive electrochemical water splitting with low power consumption, efficient, durable and affordable electrocatalysts with low overpotentials towards the hydrogen evolution reaction (HER)^4^ and oxygen evolution reaction (OER)^5^ are in urgent need.^6^ In particular, the OER remains a bottleneck for EWS owing to its sluggish kinetics.^7^ Although attempts have been made to use earth abundant transition metals^8^ and their corresponding compounds^9^ as electrocatalysts to reduce cost,^10^ their poor stability, excessive load and high overpotential partially meet the requirements of practical applications.^11^ On the other hand, noble metals such as platinum (Pt),^12^ iridium (Ir),^13^ ruthenium (Ru)^14^ and their corresponding oxides^15^ are used as benchmark electrocatalysts towards EWS, given their high efficiency and stability. Nevertheless, high cost and low abundance greatly hinder the further application of noble metal electrocatalysts.^16^ Therefore, it is desired to develop low-cost EWS catalysts exhibiting both dramatic catalytic performance and stability, and cost-effectiveness for large-scale industrial production.

Recently, it has been reported that electrochemical modification can be used to prepare highly dispersed noble metal modified carbon-based electrocatalysts.^12^ This method eliminates the need to overcome excessive surface free energy of highly dispersed noble metal atoms as the traditional pyrolysis method does.^17^ Moreover, a small quantity of noble metal ion containing solution is required.^18,19^ Since Pt dissolves in sulfuric acid at 1.1 V *vs.* RHE,^20^ the modification can be readily realized at ambient temperature and pressure.^21^

In this work, we fabricate a NiFeCuPt embedded carbon matrix uniformly dispersed on NF, denoted as NFFeCuPt hereafter, using a one-step electrochemical co-deposition method. The prepared electrode exhibits a low Pt load (*ca.* 28 μg cm^−2^, 1/5 of that in PtC) and low overpotentials at 65/98 mV, 273/328 mV, and 315/405 mV to drive a current density of 100 mA cm^−2^ for the HER, the OER and EWS in acidic (0.5 M H_2_SO_4_)/alkaline (1 M KOH) solutions, respectively, ranking it as one of the most efficient EWS catalysts reported to date.^22^ Furthermore, a strategy has also been developed to modify the prepared NF-based electrode to catalyze the HER under acidic conditions (0.5 M H_2_SO_4_) with long-term stability (>750 h). Semi *in situ* X-ray photoelectron spectra (XPS) were recorded to understand the formation process of a NiFeCuPt carbon matrix on NF. This controllable, simple and low-cost electrochemical modification method is expected to be a general method for preparing highly dispersed noble metal materials as EWS catalysts having high efficiency and desired stability.

## Results and discussion

**Fig. 1:**
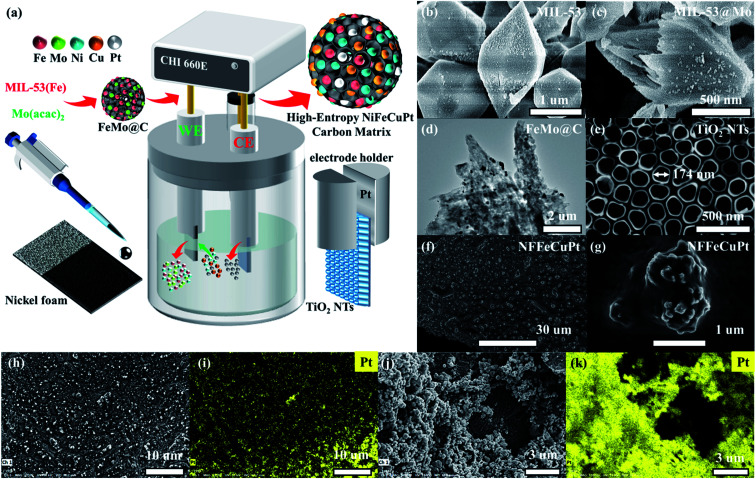
Synthesis and surface morphology of NFFeCuPt and NFFeCu–Pt electrocatalysts. (a) Schematic illustration for the preparation of a NFFeCuPt trimetallic electrode. SEM images of (b) MIL-53 and (c) MIL-53@Mo; (d) TEM images of FeMo@C; SEM images of (e) TiO_2_-NTs, (f) NFFeCuPt and (g) NFFeCuPt with higher magnification; (h, j) FESEM image of NFFeCuPt and NFFeCu–Pt as well as (i, k) corresponding EDS mapping of the Pt element.

As schematically illustrated in Fig. 1a, NFFeCuPt was synthesized by using a three-electrode system. First, a mild hydrothermal process was carried out to synthesize MIL-53 (Fe) and yield uniform fusiform particles (Fig. 1b and S1a, b, ESI†). Then MIL-53 (Fe) and C_10_H_14_MoO_6_ were mixed in a methanol solution to obtain Mo doped MIL-53 (MIL-53@Mo) (Fig. 1c and S1c, ESI†). Finally, MIL-53@Mo was carbonized in a N_2_ atmosphere to obtain FeMo bimetallic carbide (FeMo@C) (Fig. 1d and S1d, S2, ESI†). It is evident that after carbonization, the metal particles are evenly dispersed throughout the entire FeMo@C. The corresponding XRD patterns are plotted and shown in Fig. S3, ESI.† The FeMo@C ink was pipetted onto NF. After natural drying, the modified NF served as the working electrode (WE), and TiO_2_ nanotubes (TiO_2_ NTs) on the surface of a Ti sheet (Fig. 1e) as the counter electrode (CE), both of which were clamped with a platinum electrode holder and immersed in 0.5 M H_2_SO_4_, 117 mg L^−1^ of CuCl_2_·2H_2_O and 30 g L^−1^ H_3_BO_3_ as the electrolyte. The electrochemical modification was carried out by using cyclic voltammetry conducted from −0.6 to 0 V with a scan rate of 100 mV s^−1^ for 10 000 cycles. During this process, all of the Mo and a fraction of Fe dissolved in acid and vacancies were created in FeMo@C, which could trap Ni, Cu and Pt atoms subsequently. And the Pt atoms were simultaneously dissolved from the Pt electrode holder into the electrolyte around the CE and then migrated to the WE area under an electric field force, and were finally co-deposited into the vacancies mentioned above with Ni and Cu atoms from electrolyte. It is assumed that in the process of electrochemical modification, the TiO_2_ NT CE creates a localized electric field that allows metal atoms, especially Pt atoms, to embed uniformly, precisely and robustly into the carbon matrix of FeMo@C (Fig. 1f–i) by forming chemical bonds, as verified by the subsequent semi-*in situ* XPS tests. If Pt foil was used as the CE rather than TiO_2_ NTs (NFFeCu–Pt), Pt particles will be randomly deposited on the surface of NF (Fig. 1j, k; and S4, ESI†) without the confined electric field, which will greatly reduce Pt atomic utilization. According to the EDS data (Fig. S5, ESI†), the content of Pt in NFFeCu–Pt is 47 wt% but it exhibits unsatisfactory catalytic performance and stability, which contributes in part to the fact that electrochemical modification is rarely used in the synthesis of catalysts. With the aid of the TiO_2_ NT CE, NFFeCuPt achieves a lower Pt load. EDS analysis (Fig. S6, ESI†) shows that the Pt content in NFFeCuPt is 4.3 wt%, which is close to the result (4.67 wt%) acquired from inductively coupled plasma (ICP) measurements. This result also shows that nearly all the Pt atoms in NFFeCuPt are distributed on the surface of the catalyst, making it more accessible to the reactants (H^+^ or OH^−^), rather than being wrapped inside the carbon matrix, resulting in invalid dead volume and a decline in the utilization rate of Pt atoms. In addition, the load of Pt in NFFeCuPt (*ca.* 28 μg cm^−2^) is about 1/5^th^ of that in commercial 20 wt% Pt/C. More importantly, the Pt element is introduced only in one-step electrochemical modification without any pre- or post-treatments. As a source of Pt, the Pt electrode holder had no significant damage even after more than 100 NFFeCuPt sheets were manufactured (Fig. S7, ESI†). The proposed electrochemical modification method is able to maximize the utilization of Pt atoms from the perspective of atomic economy.^23^

**Fig. 2:**
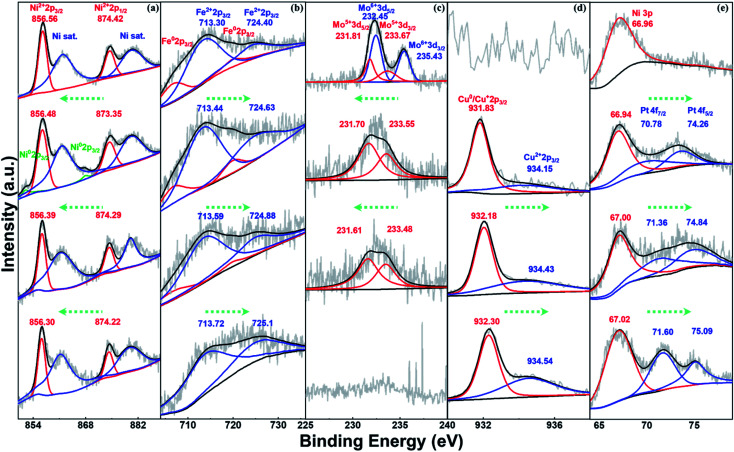
Semi *in situ* X-ray photoelectron spectra for (a) Ni 2p, (b) Fe 2p, (c) Mo 3d, (d) Cu 2p and (e) Pt 4f. From top to bottom, each image contains 4 XPS results corresponding to NFFeCuPt before CV and after CV for 3000, 5000 and 8000 cycles.

To investigate the formation process of NFFeCuPt during electrochemical modification, semi *in situ* XPS was applied to NFFeCuPt after different cycles of CV. As shown in Fig. 2a, two peaks of Ni 2p at 856.56 and 874.42 eV are ascribed to Ni^2+^ 2p_3/2_ and Ni^2+^ 2p_1/2_, respectively, along with two satellite peaks located respectively at 861.88 and 880.28 eV.^24^ The binding energy of Ni^2+^ is higher than that of the reported Ni–N_*x*_ sites^25^ which suggests that Ni exists mainly in the form of nickel oxide due to surface oxidization. With the increment in the cycles, nickel oxide is reduced at the cathode to form some elemental Ni which is then dissolved by sulfuric acid and the peaks disappear. Finally, nickel oxide on the surface gradually becomes elemental Ni and dissolves in sulfuric acid to form Ni^2+^ ions, which are adsorbed around the working electrode due to electrostatic action and then captured by the FeMo@C matrix under the reduction potential. The binding energy of Ni^2+^ decreases as the cycles of CV go up, indicating the transition from Ni–O to Ni–C bonds.^26^ As the cycles of CV go up, the unstable free elemental Fe in FeMo@C slowly dissolve out since the peaks of Fe^0^ gradually vanish (Fig. 2b). Besides, the binding energy of Fe^2+^ keeps increasing, indicating that some originally unstable Fe^2+^ is reduced and dissolved. Eventually only stable Fe–C is retained in the carbon skeleton (Fig. S8, ESI†). This not only creates vacancies and defects for other metals to fill in, but also greatly increases the proportion of stable iron active centers. According to Fig. 2c, the binding energy of Mo 3d_3/2_ and Mo 3d_5/2_ is 235.43 eV and 232.45 eV for Mo^6+^ in MoO_3_ while the peaks at 233.67 and 231.81 eV can be ascribed to Mo^5+^.^27,28^ As the cycles of CV go up, MoO_3_ will first be destroyed and reduced to Mo^5+^. Then the binding energy of Mo^5+^ decreases gradually, indicating that Mo atoms slowly escape from the matrix lattice of FeMo@C. Eventually, Mo and its compounds dissolve completely in acidic electrolyte, which are also undetectable in ICP. In general, the steady dissolution of Mo and Fe creates many lattice defects and dredges the previously blocked pore structures for Ni, Cu and Pt atoms to anchor (Fig. S9 and S10, ESI†). As shown in Fig. 2d, the Cu 2p_3/2_ peaks appearing after 3000 cycles show two deconvoluted peaks at 931.83 and 934.15 eV, corresponding to Cu (0) or Cu(i) and Cu(ii) species.^29,30^ This binding energy is smaller than the reported binding energy of the Cu-N bond,^31^ so it can be regarded as the Cu–C bond generated by Cu embedding into the FeMo@C matrix in the process of cathode deposition.^32^ Subsequently, the binding energy and the ratio of Cu^2+^ increase as the number of cycles goes up, which indicates that Cu atoms are continuously embedded in the skeleton throughout the cyclic voltammetry process. In addition, unlike Ni, Fe and Mo, the peak of Cu (0) finally exists, indicating that Cu ions form a very thin layer on the surface due to the reduction current. As shown in Fig. 2e, the Pt is not detected before CV, and only the Ni 3p peak can be observed which can be attributed to the NF base. After CVs for 3000 cycles, the peaks at 70.78 eV and 74.26 can be assigned to Pt 4f_7/2_ and Pt 4f_5/2_,^33^ which proves that the platinum sheet inside the electrode holder is indeed dissolved from the CE. And the Pt atoms migrate to the WE under the electric field force and are finally deposited on the surface of the WE.^34^ Compared with some existing literature studies,^21,35^ the binding energies shown in Fig. 2e demonstrate that Pt atoms are embedded in the carbon matrix in the form of Pt–C bonds rather than being dissociated in the pore structure of FeMo@C in the elemental form. This enhances the stability of Pt atoms, and the ligand effect can be used to regulate the adsorption energy of Pt towards H^+^ or OH^−^,^36^ which can greatly improve the performance of the catalyst. Meanwhile, the binding energy of Pt in NFFeCuPt is higher than that of Pt/C (Fig. S11a, ESI†). In addition, the peaks of Pt 4f in commercial Pt/C are sharper relative to the baseline, indicating a higher platinum content in commercial Pt/C than in NFFeCuPt. Last but not the least, no Ti 2p signal is detected by XPS (Fig. S11b, ESI†), indicating that Ti is not deposited on the WE. In fact, TiO_2_ NTs are quite stable at the operating voltage in this work. The surface of TiO_2_ NTs undergoes more than 100 electrochemical modification processes and the nanotube structure is still clear and visible (Fig. S12, ESI†).

**Fig. 3:**
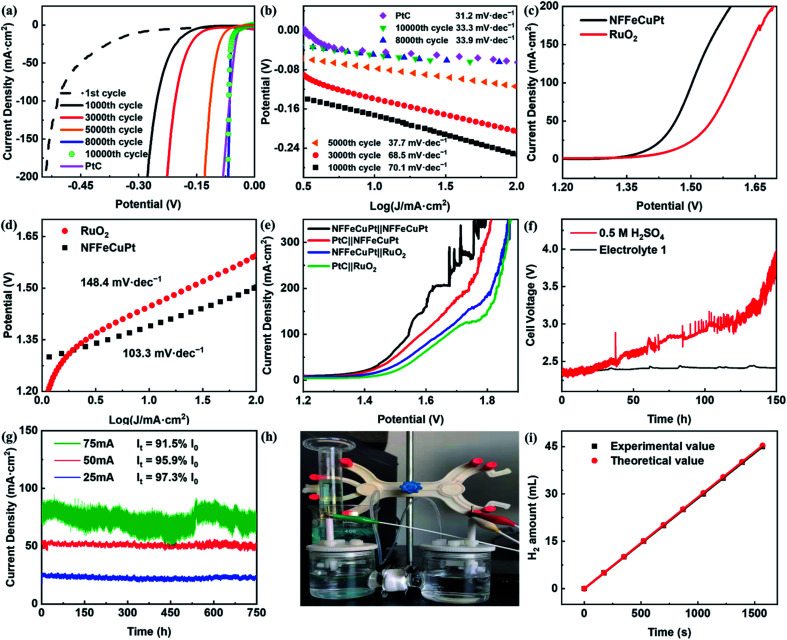
Electrochemical performance of NFFeCuPt for the HER, the OER and EWS in an acidic environment (0.5 M H_2_SO_4_). (a) LSV polarization curves after different CVs and (b) the corresponding Tafel plots; (c) OER LSV curves and (d) the corresponding Tafel plots; (e) EWS LSV curves; (f) chronopotentiometric tests of NFFeCuPt at 100 mA cm^−2^ in different electrolytes; (g) potentiostatic tests of NFFeCuPt at different current densities in Electrolyte 1; (h) home-made drainage device; (i) Faraday efficiency of NFFeCuPt.

The HER catalytic performance was first tested in 0.5 M H_2_SO_4_. As shown in Fig. 3a, with the increase of the CV cycles, the HER overpotentials of NFFeCuPt gradually decrease. As a comparison, different CEs and WEs were applied for the electrochemical modification process (Fig. S13, ESI†), but the performances were not as satisfactory as that of NFFeCuPt, indicating that the confining electric field provided by the TiO_2_ NT CE, the dissolution–redeposition process of NF and the dissolution of Mo compounds are crucial to the catalytic performance. Although PtC shows a lower HER overpotential (33 mV) at 10 mA cm^−2^ compared to NFFeCuPt (40 mV), the latter exhibits a lower overpotential when the current density is greater than 100 mA cm^−2^. This is because Cu, as a weak adsorption element towards H, can effectively promote the desorption step of hydrogen, making Pt catalytic sites in NFFeCuPt less susceptible to poisoning at high current density than PtC.^31^ To gain insight into the kinetics and mechanism of the HER, the Tafel plots derived from the corresponding polarization curves are presented in Fig. 3b, in which the Tafel slope of NFFeCuPt (33.3 mV dec^−1^) is close to that of PtC (31.2 mV dec^−1^), indicating that the Tafel step is the rate determining step.^37^ The lower Tafel slope shows better HER kinetics.^38^ In an acidic electrolyte, H^+^ obtains an electron to form H atoms and adsorbed on the surface of NFFeCuPt (Volmer reaction, H_3_O^+^ + е^−^ → H_ads_),^39^ followed by either an electrochemical desorption step (Heyrovsky step, H_ads_ + H_3_O^+^ + е^−^ → H_2_) or a chemical desorption step (Tafel step H_ads_ + H_ads_ → H_2_) to form H_2_.^40^ The theoretical Tafel slopes of the Volmer, Heyrovsky and Tafel reactions are −120, −40 and −30 mV dec^−1^, respectively.^41^ In comparison, the Tafel slope of NFFeCuPt is 33.3 mV dec^−1^ which demonstrates a Volmer–Tafel mechanism with the Tafel step as the rate determining step. A plausible mechanism of the HER under acidic conditions can be described as follows: H^+^ obtained an electron provided by the WE and adsorbed on the surface of NFFeCuPt (Volmer reaction, H_3_O^+^ + е^−^ → H_ads_), and then two H atoms adsorbed by two adjacent active sites are chemically desorbed to form hydrogen (Tafel step H_ads_ + H_ads_ → H_2_) which is finally released from the surface of the catalyst. The electrochemical impedance spectra (EIS) were used to elucidate the charge transfer resistances of NFFeCuPt. NFFeCuPt has the lowest intrinsic resistance and charge transfer resistance among the other control groups (Fig. S14, ESI†), which facilitates the kinetics towards the HER.^42^

To examine the bifunctional EWS catalytic activity, the electrocatalytic OER performance of NFFeCuPt and RuO_2_ catalysts^43^ was also measured in 0.5 M H_2_SO_4_. As shown in Fig. 3c and d, NFFeCuPt (273 mV, 103.3 mV dec^−1^) exhibits a lower overpotential at 100 mA cm^−2^ and a smaller Tafel slope than RuO_2_ (367 mV, 148.4 mV dec^−1^), suggesting that NFFeCuPt is more favorable than RuO_2_ to catalyze the OER kinetically.^44^ Next, different electrode pairs were assembled and their EWS performance was tested. As shown in Fig. 3e, it can be found obviously that the NFFeCuPt‖NFFeCuPt pair possesses the best EWS performance of all, which reduces the overpotential by 128 mV at 100 mA cm^−2^ current density compared to the commercial PtC PtC‖RuO_2_ pair. However, NF was not a desirable supporter under acidic conditions especially as an anode. The NF would dissolve (Fig. S15, ESI†) when the oxidation potential was applied (this is also why the curve of current density fluctuates obviously when the voltage increases to a certain value as shown in Fig. 3e), and so, it is impossible for NF to serve as an anode for long-term electrolysis. As far as we know, Cu is an element that is often used for underpotential deposition on the surface of other noble metals to form dense monatomic layers for measuring electrochemical surface area.^45^ Inspired by this, we tried to protect the active sites of NFFeCuPt by electroplating a layer of Cu on the electrode. Therefore, a peculiar electrolyte has been developed that allows NFFeCuPt to stably catalyze the HER under acidic conditions. The electrolyte contained 0.5 M H_2_SO_4_, 117 mg L^−1^ of CuCl_2_·2H_2_O, 30 g L^−1^ of NaH_2_PO_2_, 30 g L^−1^ of C_6_H_5_Na_3_O_7_ and 30 g L^−1^ of H_3_BO_3_, and was named Electrolyte 1. It can be seen in Fig. 3f that NFFeCuPt exhibits worse stability in 0.5 M H_2_SO_4_ than in Electrolyte 1. This is probably because the Cu^2+^ in Electrolyte 1 can form a stable layer on the cathode surface due to the electric field force (Fig. S16, ESI†), which can protect the NF from being corroded by sulfuric acid.

To prove that Electrolyte 1 can help NFFeCuPt achieve enough stability for practical application, a potentiostatic test was carried out for NFFeCuPt in Electrolyte 1 with the initial current densities at 25 mA cm^−2^, 50 mA cm^−2^ and 75 mA cm^−2^. It can be seen clearly in Fig. 3g that the current density loss is inconspicuous even after working for 750 h, which is a breakthrough for the stability of NF under acidic conditions.^46,47^ In addition, with the increase of current density, the Cu film may become denser (Fig. S17, ESI†) due to a higher applied potential, preventing some active sites from accessing the reactants, which is also the reason for more current density loss. Since graphite rods will dissolve after serving as a CE for about 300 h (Fig. S18, ESI†), the WE (NFFeCuPt) and CE (RuTiIr) were connected to a copper wire to assemble a cell for a long-term stability test (Fig. S19, ESI†). The generated H_2_ gas was qualified by using a home-made drainage device (Fig. 3h)^48^ and a FE of 99.5 ± 1% is obtained (Fig. 3I), indicating that the formed Cu film has little effect on FE. Next, galvanostatic and potentiostatic (Fig. S20, ESI†) measurements were carried out to investigate the stability of NFFeCuPt at high current density. Nevertheless, when the current density of the galvanostatic test exceeded 300 mA cm^−2^, there would be a noticeable decline after electrolysis for about 50 h (Fig. S21, ESI†). Interestingly, when the electrolyte solution was renewed, the current density can reach the initial value (Fig. S22, ESI†), which means that NFFeCuPt has not been damaged. This is probably due to the dissolution of the NF substrate at high current density (Fig. S23, ESI†). The SEM images of NFFeCuPt (after the potentiostatic test in Electrolyte 1) before and after the H_2_SO_4_ treatment (Fig. S24, ESI†) show the presence of a copper film. Notably, as the CE was no longer TiO_2_ NTs during the stability test, the carbon matrices on NF tended to agglomerate after the stability test (Fig. S24c and d, ESI†), which also proves the existence of the confining electric field effect of TiO_2_ NTs during the synthesis process of NFFeCuPt. Finally, the ECSA is estimated by using CVs to compare the intrinsic catalytic activity of NFFeCuPt (Fig. S25, ESI†). Although NFFeCu–Pt has a larger ECSA and a greater number of potential active sites, NFFeCuPt exhibits a much better catalytic performance (Fig. S13, ESI†), indicating that the active sites of the latter have higher intrinsic activity. Furthermore, due to the disordered aggregation of Pt (Fig. 1h and i) and excessive adsorption capacity of the H^+^ intermediate, Pt sites in NFFeCu–Pt are poisoned by H^+^ in the early stages of electrolysis (Fig. S26, ESI†) while NFFeCuPt has a stable catalytic activity due to the Cu sites promoting the desorption step.^32^ In conclusion, there are two major advantages of the electrochemical modification in this work. First, it can greatly improve the utilization rate of platinum atoms since it can construct catalytic sites with higher intrinsic activity compared to traditional methods. Secondly, it can simultaneously introduce Cu atoms, which are conducive to the H_2_ desorption step, into the carbon matrix by one-step co-deposition to avoid the Pt catalytic sites being poisoned by excessive adsorption of H^+^ under acidic conditions at high current density during the long-term electrolysis process.

**Fig. 4:**
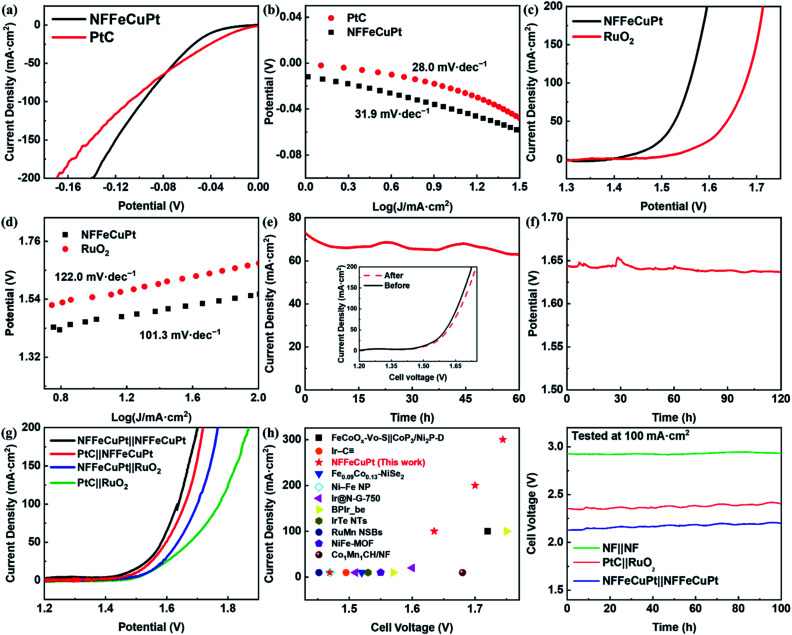
Electrochemical performance of NFFeCuPt for the HER, the OER and EWS in an alkaline environment (1 M KOH). (a, c) LSV polarization curves; (b, d) corresponding Tafel plots; (e) potentiostatic test of NFFeCuPt at 1.624 V and the inset is the LSV curves before and after the potentiostatic test; (f) chronopotentiometric tests of NFFeCuPt at 100 mA cm^−2^; (g) EWS LSV curves; (h) comparison of the overall water splitting activity in this work and other works; (i) chronopotentiometric tests of EWS at 100 mA cm^−2^ in 1 M KOH.

Since NF is not capable of catalyzing long-term OERs under acidic conditions, the EWS performance of NFFeCuPt under alkaline conditions (1 M KOH) is comprehensively evaluated to demonstrate its versatility. First, the overpotentials needed to achieve current densities of 10 mA cm^−2^ and 100 mA cm^−2^ are 39 mV and 97 mV for NFFeCuPt and 25 mV and 102 mV for PtC (Fig. 4a), which once again suggest that Cu atoms in NFFeCuPt can promote desorption steps to improve catalytic performance at higher current density. This can also be confirmed from the Tafel plots in Fig. 4b. Although in the range of small current density, PtC has a smaller Tafel slope (28.0 mV dec^−1^) compared with NFFeCuPt (31.9 mV dec^−1^), with the increase of current density, the Tafel slope of PtC tends to increase while that of NFFeCuPt does not. It is worth noting that the mechanism of the HER under alkaline conditions is slightly different from that under acidic conditions. Unlike the acidic condition which is rich in H^+^, under alkaline conditions, the dissociation of water (H_2_O → H^+^ + OH^−^) has a great impact on the HER since it is considered as the largest source of H^+^.^49^ Therefore, even the Tafel slope of NFFeCuPt is 31.9 mV dec^−1^ in 1 M KOH which is a Volmer–Tafel mechanism with the Tafel step as the rate determining step. The HER under alkaline conditions should proceed in this way: First, the dissociation of water (H_2_O → H^+^ + OH^−^) occurred in the catalytic sites to provide H^+^ for further use. Then the Volmer reaction (H_2_O + е^−^ → H_ads_ + OH^−^) and the Tafel step (H_ads_ + H_ads_ → H_2_) on the active sites.^50^

Next, the OER performance was also tested in 1 M KOH. NFFeCuPt (230 mV to 10 mA cm^−2^ and 330 mV to 100 mA cm^−2^) exhibited a lower overpotential (Fig. 4c) compared to RuO_2_ (310 mV to 10 mA cm^−2^ and 440 mV to 100 mA cm^−2^) and favorable kinetics with a lower Tafel slope (Fig. 4d). Additionally, according to some previous reports,^51,52^ the voltage and current required for *in situ* oxidation of nickel foam to form a layer of nickel oxide are far lower than the conditions for the OER to occur. Since the onset potential of the OER is at least 1.23 V *vs.* RHE, at this potential, in an alkaline, oxygen-rich environment, it can be reasonably assumed that oxygen atoms are inserted into the nickel lattice (Fig. S27, ESI†). Fortunately, the oxygen atoms inserted into the nickel lattice on the surface of NFFeCuPt can assist in the dissociation of water under alkaline conditions (H_2_O → H^+^ + OH^−^)^53,54^ which has a great impact on the EWS reaction^49^ since the oxygen atoms inserted into the nickel lattice form new and independent hydrolytic dissociation actives site.^55^ These dissociation sites can promote water dissociation (H_2_O → H^+^ + OH^−^, non-redox process) alone with the NFFeCuPt active sites facilitating the OER (4OH^−^ + 4e^−^ → O_2_ + H_2_O, redox process) respectively.^56^ In conclusion, the mechanism of the OER under alkaline conditions can be described as follows:^57^ the OH^−^ loses an electron and adsorbs on the active sites of the WE (M + OH^−^ → MOH + e^−^). Then the absorbed MOH intermediate loses an electron step by step and is converted into other oxygen-containing intermediates (MOH + OH^−^ → MO + H_2_O + e^−^; MO + OH^−^ → MOOH + e^−^) and finally the MOOH intermediate loses an electron and releases O_2_ (MOOH + OH^−^ → M + O_2_ + H_2_O + e^−^). Moreover, the potentiostatic and chronopotentiometric test results in Fig. 4e and f show that NFFeCuPt has excellent stability under alkaline conditions towards the OER. For comparing the performance of different electrode pairs towards overall water splitting under alkaline conditions, a comparison was made between different catalysts supported by NF. It can be concluded as shown in Fig. 4g that in order to achieve a current density of 100 mA cm^−2^, cell voltages of 1.638 mV, 1.659 mV, 1.701 mV and 1.772 mV need to be applied to the following four electrode pairs including NFFeCuPt‖NFFeCuPt, NFFeCuPt‖RuO_2_, PtC‖NFFeCuPt and PtC‖RuO_2_, respectively. The overall water splitting performance of NFFeCuPt is superior to most of the other catalysts reported recently especially at high current density, including FeCoO_*x*_–Vo–S‖CoP_3_/Ni_2_P-D,^48^ Ir–C≡,^22^ Fe_0.09_Co_0.13_–NiSe_2_,^15^ Ni–Fe NP,^58^ Ir@N-G-750,^59^ BPIr_be,^60^ IrTe NTs,^61^ RuMn NSBs,^62^ NiFe-MOF,^63^ Co_1_Mn_1_CH/NF^64^ and so on (Fig. 4h). As for Pt in NFFeCuPt, the mass-specific activity is 848.9 A g^−1^ at an overpotential of 300 mV. Meanwhile, a two-electrode system was used to evaluate the stability of NFFeCuPt at a current density of 100 mA cm^−2^ using the galvanostatic method in 1 M KOH (Fig. 4i), indicating that the NFFeCuPt‖NFFeCuPt pair has not only comparable stability as commercial PtC‖RuO_2_, but also a lower overpotential towards EWS, which well meets the requirement of practical water splitting. In addition, the electrochemical performances of NFFeCuPt for the HER, the OER and EWS in a neutral environment (0.1 M PBS) were also recorded (Fig. S28, ESI†). Although the performances of NFFeCuPt exceed those of commercial noble-metal materials and are comparable to recently reported EWS materials,^60^ the high overpotentials (*η*_10_ = 369 mV for the HER; *η*_10_ = 684 mV for the OER; *η*_10_ = 1379 mV for EWS) still hinder the practical application of NFFeCuPt under neutral conditions. Last but not least, OER stability of NFFeCuPt was verified in a highly alkaline environment (Fig. S29, ESI†) at a higher current density (Fig. S30, ESI†). The extreme alkaline conditions can be applied in the OER during alkaline zinc–air battery charging.^65^

## Conclusions

In conclusion, the synthesized NFFeCuPt catalyst exhibited superior electrocatalytic performance and stability for water splitting in acidic, neutral and alkaline environments. The proposed electrochemical modification demonstrated in this study can greatly improve the utilization rate of precious materials by accelerating the intrinsic activity of catalytic active sites and reducing the waste of noble metal atoms caused by redundant steps in traditional synthesis methods. Furthermore, the common approach used in this work can greatly improve the stability of a NF supporter towards the HER under acidic conditions. Such a cost-effective, simple and environmentally friendly electrochemical modification strategy provides alternative insights for fabricating low-load noble metal electrocatalysts with high activity and satisfactory stability, which can also be extended to the synthesis of various electrocatalytic materials at room temperature due to its desirable controllability.

## Data availability

All the calculation data and experimental data reported in this paper could be accessed from the authors.

## Author contributions

Ziqi Zhang: conceptualization, investigation, methodology, software, and drafting of the manuscript. Yiduo Li: Investigation and software. Zhe Zhang: software and formal analysis. He Zheng: analysis. Yuxin Liu: visualization and methodology. Yuxing Yan: visualization and methodology. Chunguang Li: conceptualization and resources. Haiyan Lu: data curation and funding acquisition. Zhan Shi: conceptualization, funding acquisition, project administration and supervision. Shouhua Feng: investigation and resources.

## Conflicts of interest

There are no conflicts to declare.

## Supplementary Material

SC-013-D2SC02845J-s001

SC-013-D2SC02845J-s002
