# Supplementary material for: An electrochemical modification strategy to fabricate NiFeCuPt polymetallic carbon matrices on nickel foam as stable electrocatalysts for water splitting
Source: Chem Sci. 2022 Jul 5;13(30):8876–84. doi: 10.1039/d2sc02845j (PMC9350615; doi:10.1039/d2sc02845j)
Supplement: SC-013-D2SC02845J-s002 [file SC-013-D2SC02845J-s002.pdf]

Electronic Supplementary Material (ESI) for Chemical Science.
This journal is © The Royal Society of Chemistry 2022
Electronic Supplementary Material (ESI) for Chemical Science.
This journal is © The Royal Society of Chemistry 2022

Electrochemical Modification Strategy to Fabricate NiFeCuPt Polymetallic Carbon Matrixes on Nickel Foam as Stable Electrocatalyst for Water Splitting

Ziqi Zhang,[a] Yiduo Li,[a] Zhe Zhang,[a] He Zheng,[a] Yuxin Liu,[a] Yuxing Yan,[a] Chunguang Li,[a] Haiyan Lu,*[a] Zhan Shi*[a] and Shouhua Feng[a]

[a] *State Key Laboratory of Inorganic Synthesis and Preparative Chemistry, College of Chemistry, Jilin University, Changchun 130012, P. R. China.*

E-mail: zshi@mail.jlu.edu.cn; luhy@jlu.edu.cn

Experimental Procedures

Materials

Iron(III) chloride hexahydrate ($FeCl_3 \cdot 6H_2O$, Aladdin), 1,4-dicarboxybenzene ($C_8H_6O_4$, Aladdin), Bis(acetylacetonato)dioxomolybdenum(VI) ($C_{10}H_{14}MoO_6$, Aladdin), 1,4-dicarboxybenzene (1,4-BDC, $C_8H_6O_4$, Aladdin), boric acid (H_3BO_3, Beijing Chemical Works), copper chloride dihydrate ($CuCl_2 \cdot 2H_2O$, Aladdin), platinum on carbon (Pt/C, 20 wt%, Tanaka Kikinzoku International KK, Japan), ruthenium(IV) oxide (RuO_2, 99.9% trace metals basis, Sigma-Aldrich), nafion (5 wt%, Dupont), trisodium citrate dihydrate ($C_6H_5Na_3O_7$, Beijing Chemical Works), sodium hypophosphite (NaH_2PO_2, Beijing Chemical Works), nickel foam (NF, Kunshan GuangJiaYuan New Materials Co. Ltd., China), copper foam (CF, Kunshan GuangJiaYuan New Materials Co. Ltd., China), titanium foam (TF, Kunshan Xingzhenghong Electronic Materials Co. Ltd, China), Ti sheet (Yitai Metal, China), hydrofluoric acid (HF, Beijing Chemical Works), nitric acid (HNO_3, Beijing Chemical Works), glycol ($C_2H_6O_2$, Beijing Chemical Works), methanol (CH_3OH, AR, Beijing Chemical Works), ethyl alcohol (C_2H_5OH, ≥ 99.8%, Beijing Chemical Works), phosphate-buffered saline solution (PBS, 0.1 M, pH=7.4, Phygene), sulfuric acid (H_2SO_4, 98%, Beijing Chemical Works) and potassium hydroxide (KOH, Beijing Chemical Works) were employed. All chemical reagents were used as received without further purification. All aqueous solutions were prepared with ultrapure water (resistivity of 18. 25 MΩ cm). Besides, platinum electrode holder, glassy carbon electrode (GCE, ⌀ = 3 mm), graphite rod electrode, Pt foil (1 × 1 cm^2) electrode, Hg/Hg_2SO_4 (saturated K_2SO_4 electrolyte) electrode, Hg/HgO (1 M KOH electrolyte) electrode, saturated calomel electrode (SCE), carbon paper and electrolytic cell were purchased from Shanghai Yue Magnetic Electronic Technology Co., Ltd. Dimensionally Stable Anodes (DSA) including Ruthenium-titanium-iridium anode (RuTiIr) and titanium selenium antimony anode (TiSeSb) were purchased Anhui Zhengying Technology Co., Ltd.

Physical Characterization

Scanning electron microscopy (SEM) was performed on a HITACHI SU020 microscope and JEOL JSM-7800F with an operating voltage of 3 kV. EDS mapping was conducted on a Thermo FEI environmental scanning electron microscope (ESEM). Transmission electron microscopy (TEM) was performed on a Philips FEI Tecnai G2S-Twin microscope equipped with a field emission gun operating at 200 kV. X-ray photoelectron spectra (XPS) were recorded on a Thermo Fisher Scientific ESCALAB 250Xi unit with Al-Kα (1486.6 eV) as an X-ray source. X-ray diffraction (XRD) patterns were carried out using a Rigaku D/MAX-2550 with Cu Kα radiation and accelerating voltage and applied current were 40 kV and 20 mA,

respectively, and the diffraction data were recorded in the 2θ range of 5–80° with a scan rate of 5° per min. XRD data were processed by JADA 6 with International Centre for Diffraction Data (ICDD-PDF) as database. Gas adsorption–desorption analyses were conducted using N_2 as adsorbent on a ASAP 2020 (Micromeritics instrument, United States) by nitrogen adsorption at 77 K. Raman spectrum was recorded on a Thermo Fisher DXRxi. Inductively coupled plasma (ICP) data were collected from Agilent 725 (Agilent Technologies Inc). Thermal gravimetric analysis (TGA) measurements were carried out on a STA 499 F3 instrument (Netzsch) under N_2 atmosphere with a heating rate of 10 °C per min.

Synthesis of MIL-53 (Fe)

MIL-53 (Fe) (MIL = Materials of Institut Lavoisier) was prepared by a mild solvothermal process [1] with some modifications. Sixteen mmol (4.339 g) of $FeCl_3 \cdot 6H_2O$ and 16 mmol (2.653 g) of 1,4-BDC were added slowly into 80 mL of DMF solution. After stirring for 10 min, the mixture was transferred into a Teflon-lined stainless steel autoclave with a volume capacity of 100 mL and heated at 160 °C for 2 h. Then the mixed solution was centrifuged and rinshed with methanol several times and dried in vacuum at 60 °C overnight to yield MIL-53 (Fe).

Synthesis of FeMo@C

Two hundred mg of MIL-53 (Fe) was dispersed in 20 mL of methanol by ultrasound, which was subsequently injected into 20 mL of methanol containing 200 mg of $C_{10}H_{14}MoO_6$. Then the mixed solution was stirred at room temperature for 24 h, centrifuged with methanol several times and dried in vacuum at 60 °C overnight to obtain MIL-53@Mo. The precipitate was heated at 800 °C for 3 h at a rate of 3 $°C \cdot min^{-1}$ in a tube furnace under N_2 atmosphere to get Fe-Mo bimetallic carbide (FeMo@C). As control groups, MIL-53 (Fe) was heated under the same conditions as above without the addition of $C_{10}H_{14}MoO_6$ to obtain corresponding carbonization products which were named as Fe@C.

Synthesis of TiO_2 nanotubes on the surface of Ti

TiO_2 nanotubes were prepared on titanium surface using the electrochemical anodization method[2]. First, 15 mm × 10 mm × 0.1 mm titanium sheet was ultrasonic washed in 10 mL of acetone, 10 mL of ethanol and deionized water for 5 min, respectively. Then titanium sheet was immersed in 10 mL of $HF:HNO_3:H_2O$ = 1:4:5 (volume ratio) mixed solution for 15 s to remove the oxide layer. Titanium sheet was clamped to the electrode holder and used as anode while platinum electrode as cathode (no reference electrode was introduced during the anodization process). The two electrodes were placed in a 250 mL electrolytic cell with a mixture of 10 ml NH_4F (0.33 g) aqueous solution and 90 mL glycol as electrolyte. After both electrodes were immersed in electrolyte, anodic oxidation process was carried out by a 60-volt

constant voltage power supply for 120 min. After anodization, sample was washed with deionized water and dried. Finally, titanium with TiO_2 nanotubes on surface was obtained and designated as TiO_2 NTs**.**

Preparation of NFFeCuPt trimetallic electrode

Electrochemical modifications and measurements were performed by a CHI 660E electrochemical workstation with a three-electrode system, including a working electrode (WE), a counter electrode (CE) and a reference electrode (RE). **All electrode potentials were relative to reversible hydrogen electrodes (RHE) unless otherwise stated.** Besides, when electrolyte was 0.5 M H_2SO_4 solution, the Hg/Hg_2SO_4 (saturated K_2SO_4 electrolyte) electrode served as reference electrode (RE) while Hg/HgO (1 M KOH electrolyte) electrode was used in 1 M KOH solution. The experimental potential values were calibrated by using the following equation: $E_{vs.\ RHE} = E_{vs.\ Hg/Hg2SO4} + 0.64 + 0.059$ pH; $E_{vs.\ RHE} = E_{vs.\ Hg/HgO} + 0.098 + 0.059$ pH. Before electrochemical modifications and measurements, the reference electrodes were all calibrated by CV tests using glassy carbon electrode as standard WE.

Ten mg of FeMo@C was dispersed in 900 μL of ethyl alcohol and 100 μL of 5 wt. % nafion mixed solution and ultrasonic for 30 min. 60 μL of liquid was pipetted onto a portion of the surface (1×1 cm^2) of NF with natural drying and flattened by a tablet press. Then the modified NF served as working electrode (loading 0.6mg·cm^2). A sheet of prepared NF and TiO_2 NTs was clamped to the platinum electrode holder served as WE and CE, respectively. RE was Hg/Hg_2SO_4 electrode and electrolyte contained 0.5 M H_2SO_4, 117 mg·L^{-1} of $CuCl_2 \cdot 2H_2O$ and 30 g·L^{-1} H_3BO_3. Electrochemical modification was carried out by using cyclic voltammetry conducted from −0.6 to 0 V with a scan rate of 100 mV·s^{-1}.for 10000 segments. Then the nickel foam based NiFeCuPt electrode (NFFeCuPt) was obtained. After cyclic voltammetry, linear sweep voltammetry (LSV) was conducted more than 10 times from 0 to −0.35 V with a scan rate of 10 mV·s^{-1} till the last two LSV curves were perfectly aligned so that WE surface reached a stable state.

Then WE (NFFeCuPt) was washed with deionized (DI) water thoroughly and dried naturally for further measurements. NFFeCu-Pt was obtained under the same conditions as above for comparison except for changing TiO_2 NTs CE into a Pt foil. NFCuPt and NF-FeCuPt were obtained under the same conditions as above for comparison except for adding no FeMo@C or changing the FeMo@C into Fe@C.

HER and OER performance measurements

The as-prepared WE (NFFeCuPt) was directly used as working electrode without further treatments. Exposed surface area of NF electrode was 1×1 cm^2. HER and OER LSV polarization curves were recorded at a scan rate of 10 mV·s^{-1} in 0.5 M H_2SO_4 (RE: Hg/Hg_2SO_4 (saturated K_2SO_4 electrolyte)) or 1 M KOH (RE: Hg/HgO (1 M KOH electrolyte)). Besides, LSV curves were also collected in 0.1 M PBS

(pH=7.4) solution with saturated calomel electrode (SCE) used as RE ($E_{RHE} = E_{SCE} + 0.241 + 0.059$ pH). **All LSV curves were measured with iR compensation at 100%.** Tafel plots were converted from the LSV data at low overpotential fitted to the Tafel equation (η=b log j+a, where η is overpotential, j is the current density, a is the intercept of the y-axis, and b is the Tafel slope)[3]. Electrochemical impedance spectra (EIS) measurement was performed at different overpotential with frequencies from 100 mHz to 100 kHz and an amplitude of 5 mV[4]. The electrochemically active surface area (ECSA) was estimated by CVs, which was tested from 0.024 to 0.124 with a scan rate varying from 10 to 100 $mV \cdot s^{-1}$ in 1 M KOH[5]. The current density was normalized to the geometrical area. Chronopotentiometric and potentiostatic tests were obtained under the same experimental setup except that the CE was RuTiIr instead of graphite rod. Notably, the long-time stability tests in acidic solution including chronopotentiometric and potentiostatic measurements were all conducted by using RuTiIr DSA as CE instead of graphite rod since the latter would be dissolved during the long-term electrolysis.

EWS performances measurements

EWS cell was assembled by employing a classical two-electrode (WE and CE) system[6]. LSV was recorded with a scan rates of 10 $mV \cdot s^{-1}$ by CHI 660E electrochemical workstation. Chronopotentiometric and potentiostatic tests were obtained by using constant current and constant voltage charging modes of Neware CT-4008T-5V6A-S1. Instead of being clamped to platinum electrode holders, WE and CE were connected to conductive copper wires and the joints were wrapped in teflon tape through epoxy resin. Faraday efficiency was tested using the drainage device (Movie S1)[7]. **All the above tests except LSV and CV were measured without iR compensation unless otherwise stated.**

Faradaic efficiency (FE) calculations

FE was estimated by comparing the experimentally measured gas volume and the theoretical one. A chronopotentiometric test was carried out at 100 mA cm^{-2} in Electrolyte 1. Theoretical FE was calculated based on the following equation: FE = $(2 \times F \times n)/(i \times t)$, where F is the Faraday constant (96485 C mol^{-1}); n is the number of moles of the produced gas (mol); i stands for the current (A); and t represents the electrolysis time (s).

Results and Discussion

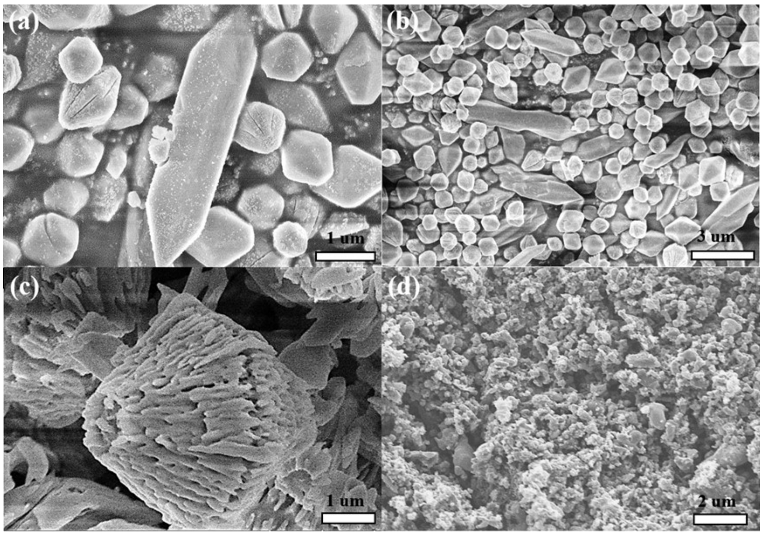

Figure S1. SEM images of (a,b) MIL-53 at different magnifications; (c) MIL-53@Mo (stirred for 36 h) ;(d) FeMo@C.

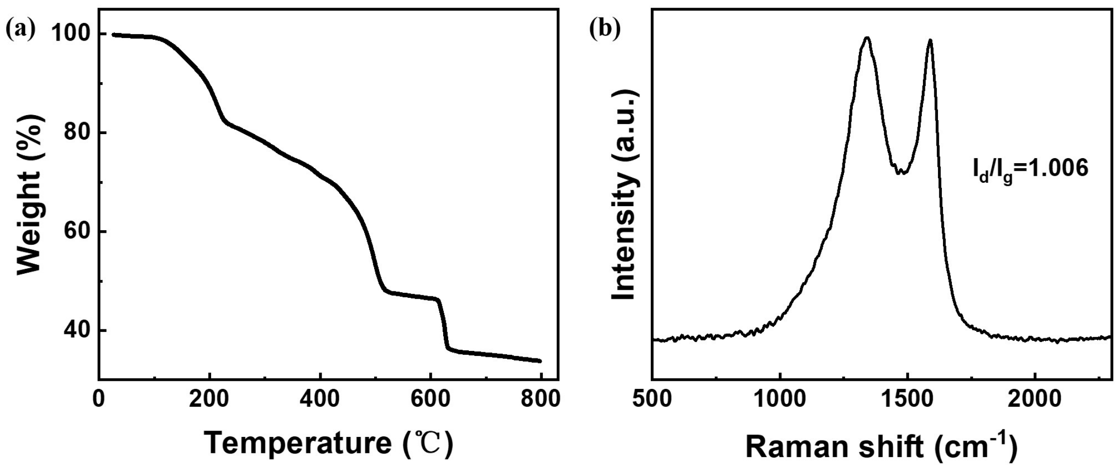

Figure S2. (a) TGA plots of MIL-53; (b) Raman spectrum of FeMo@C.

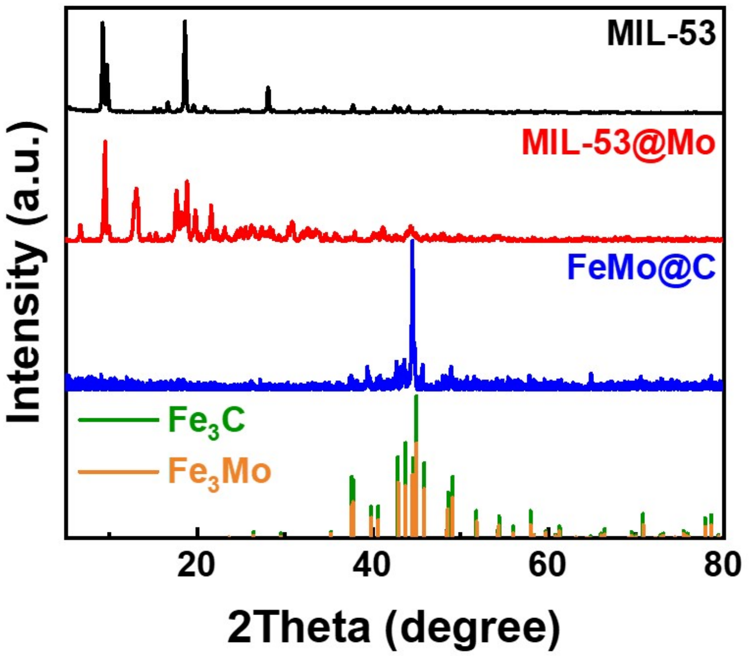

Figure S3. XRD patterns of MIL-53, MIL-53@Mo, FeMo@C, corresponding standard diffraction peaks of Fe_3C (JCPDS No: 35-0772) and Fe_3Mo (JCPDS No:31-0641).

Notes for Figure S3. MIL-53 (Fe) was synthesized since the characteristic peaks were consistent with previous literature[8]. After doping of Mo, the main skeletal structure of MIL-53 remained because the initial morphology was retained (Figure 1b) and all characteristic peaks were preserved (Figure S6, Supporting Information). Then carbonization at 800 °C converted MIL-53@Mo into a graphitized conductive skeleton Fe–Mo bimetallic carbide (Figure S7, Supporting Information) with Fe_3C and Fe_3Mo embedded in.

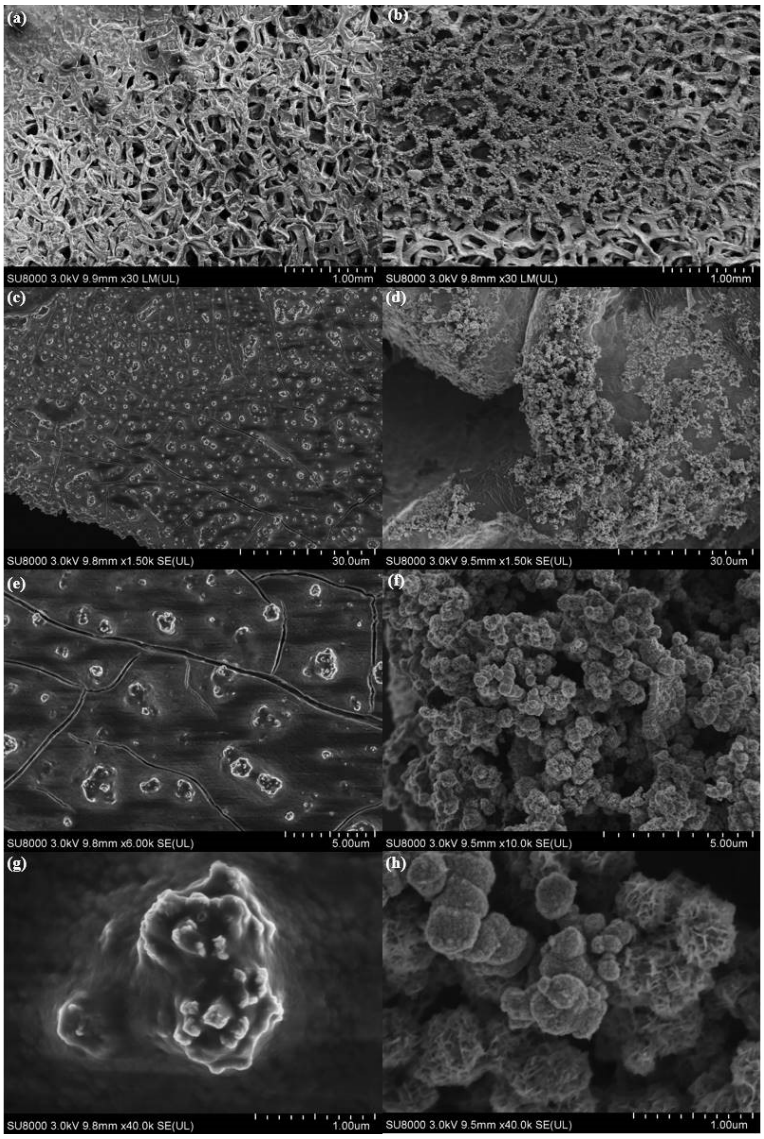

Figure S4. SEM images of (a,c,e,g) NFFeCuPt and (b,d,f,h) NFFeCu-Pt at different magnifications.

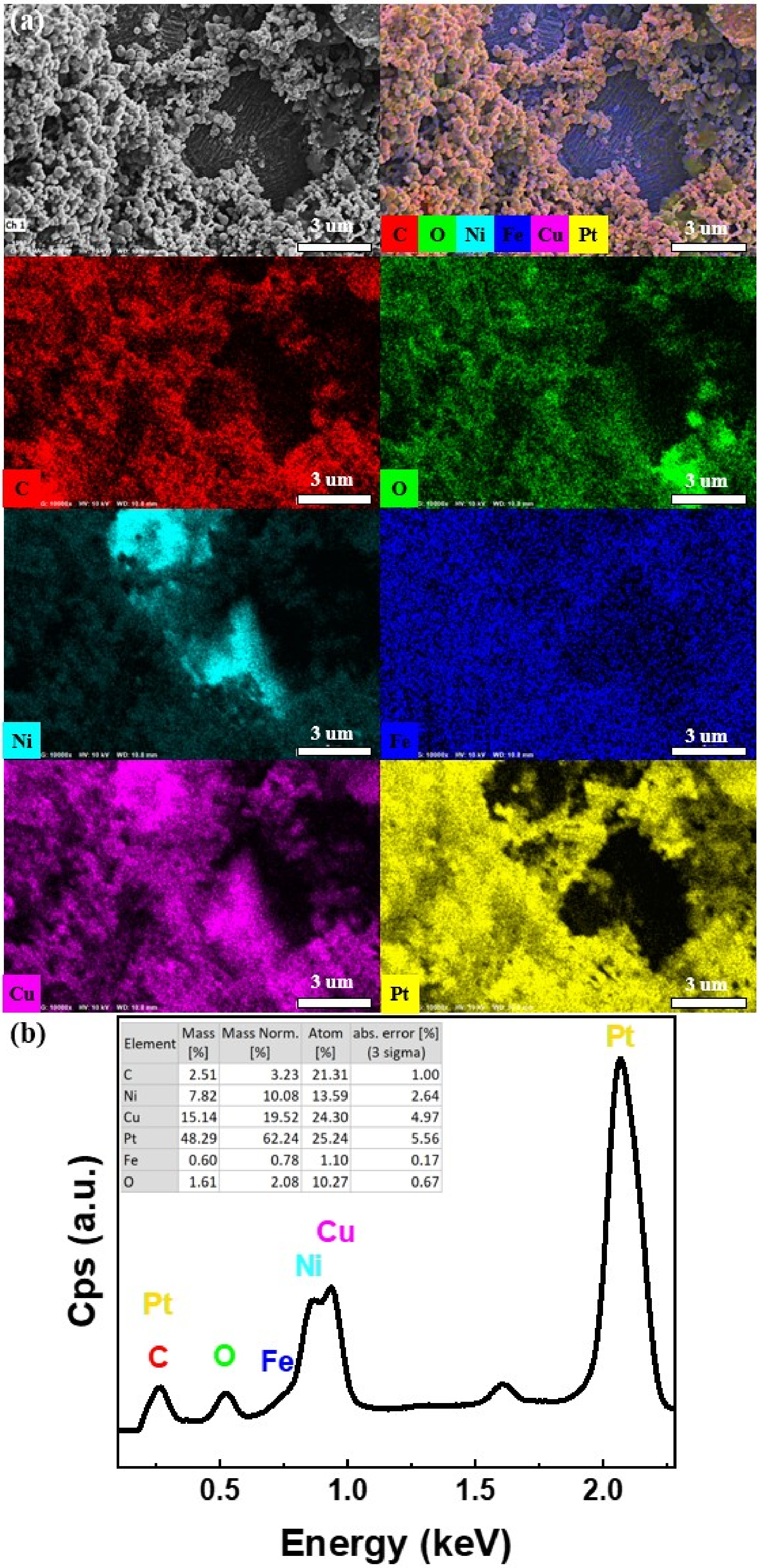

Element	Mass [%]	Mass Norm. [%]	Atom [%]	abs. error [%] (3 sigma)
C	2.51	3.23	21.31	1.00
Ni	7.82	10.08	13.59	2.64
Cu	15.14	19.52	24.30	4.97
Pt	48.29	62.24	25.24	5.56
Fe	0.60	0.78	1.10	0.17
O	1.61	2.08	10.27	0.67

Figure S5. ESEM EDS mappings (a) and corresponding EDS spectrum (b) of NFFeCu-Pt.

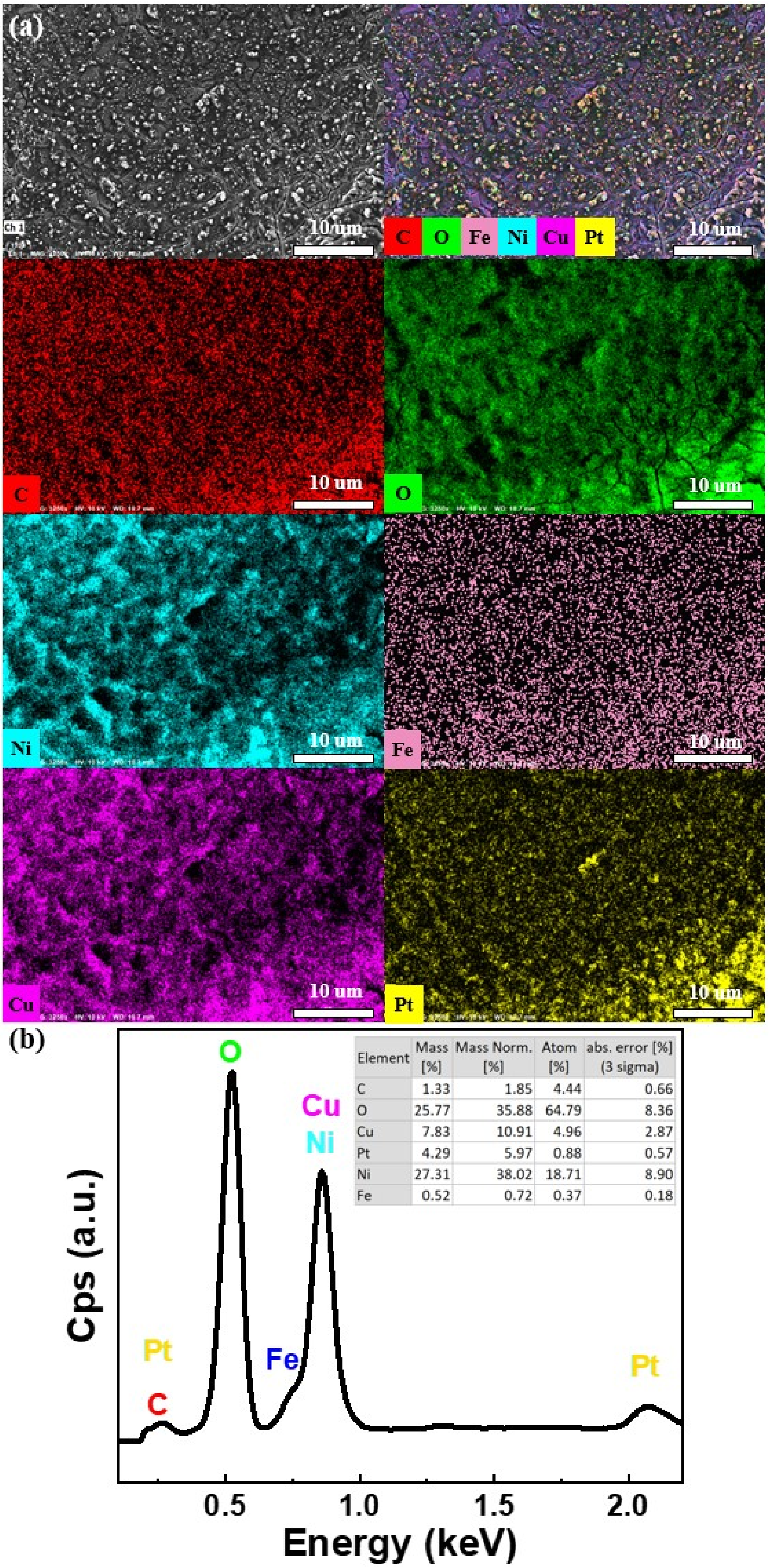

Figure S6. ESEM EDS mappings (a) and corresponding EDS spectrum (b) of NFFeCuPt.

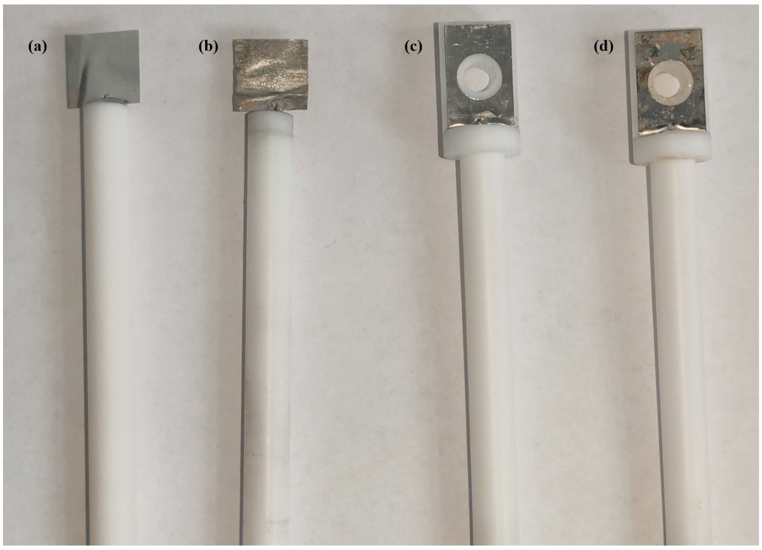

Figure S7. Optical pictures of Pt foils CEs before (a) and after (b) being used for manufacturing two sheets of NFFeCu-Pt; Pt electrode holders CEs before (c) and after (d) being used for manufacturing 100 sheets of NFFeCuPt (d).

Notes for Figure S7. In addition, the electrochemical modification electrolyte can be recycled and used for the subsequent NFFeCuPt electrodes. In the subsequent 100 times of modification, at least 70 times of the same or better performance than the prototype were tested in our laboratory to ensure reproducibility. Because same voltage parameters were applied by each electrochemical modification, the repeated use of the electrolyte can keep Pt content in electrolyte in an equilibrium state, and inhibit further dissolution of Pt of CE. On the other hand, because titanium dioxide instead of platinum is directly exposed to an acidic electrolyte, which can physically avoid electrochemical corrosion of Pt. This electrochemical method can save Pt consumption at source.

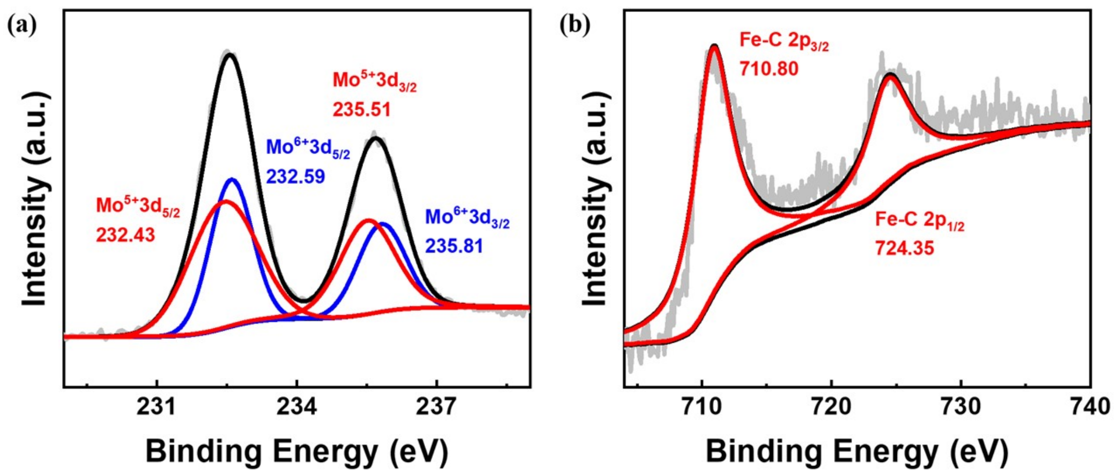

Figure S8. High-resolution XPS spectra of Mo3d (a) and Fe2p (b) of FeMo@C.

Notes for Figure S8. XPS was carried out to investigate the chemical composition and element bonding configurations of FeMo@C. The Mo 3d peaks at 232.59 eV and 235.81 eV are assigned to Mo^{6+} $3d_{5/2}$ and Mo^{6+} $3d_{3/2}$ of MoO_3 produced by the thermal decomposition of $C_{10}H_{14}MoO_6$[9]. Mo^{5+} peaks (232.43 eV and 235.51 eV) reveal the formation of oxygen vacancies due to the substitution of Fe atoms for Mo atoms in MoO_3 lattice[10] or Mo atoms in MoO_3 reduced by carbon during the thermal reduction process[4]. Figure S8b also demonstrates the formation of typical Fe-C bonds[11] during the carbonization process.

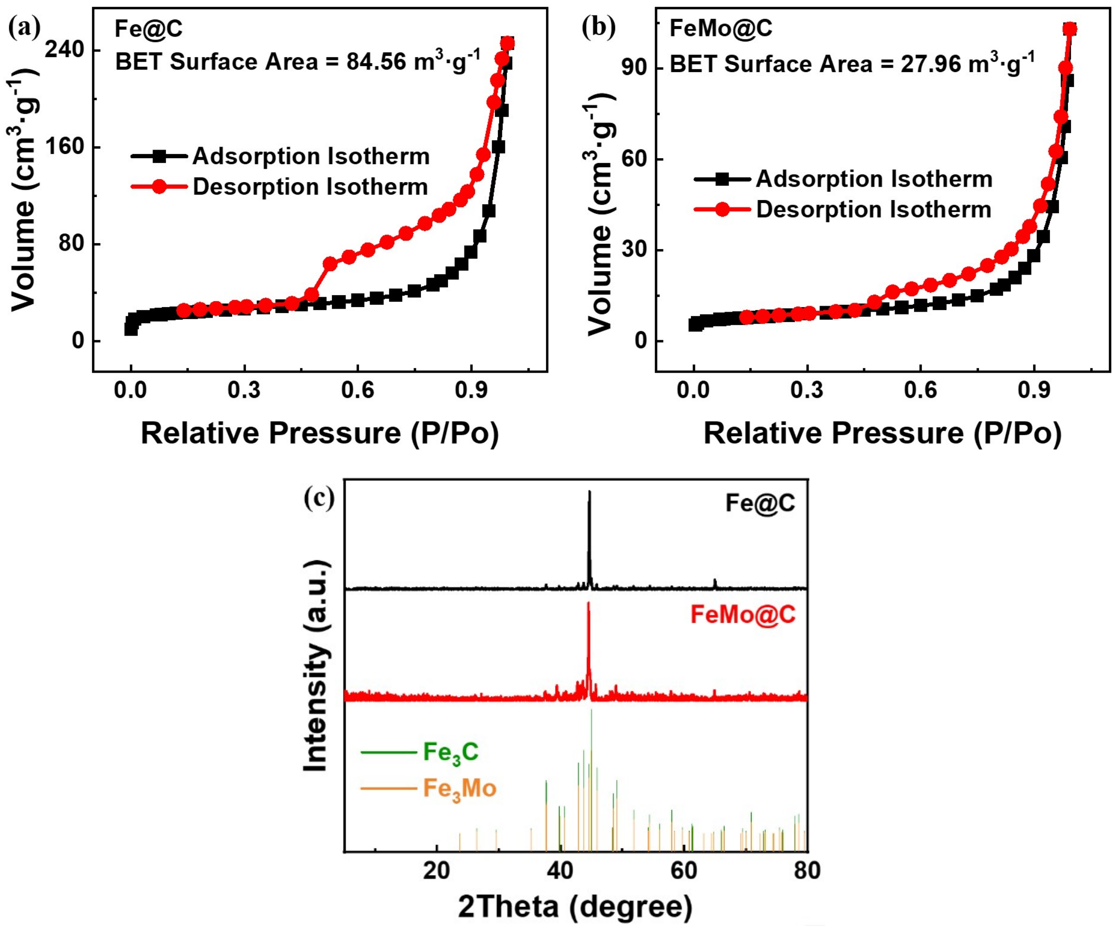

Figure S9. Nitrogen adsorption–desorption isotherms of Fe@C (a), FeMo@C (b) and corresponding XRD patterns (c).

Notes for Figure S9. N_2 adsorption–desorption was also carried out for Fe@C and FeMo@C. The typical shape of the isotherms exhibits a type IV curve with an H4-type hysteresis loop, suggesting that micro/meso-pores were oriented in Fe@C and FeMo@C[12]. Notably, the calculated Brunauer–Emmett–Teller (BET) surface area of FeMo@C was about 27.96 $m^2 g^{-1}$, which was much smaller than that of Fe@C. This indicates that doping of Mo and corresponding Fe and Mo compounds blocked part of porous structure of MIL-53 (Fe) carbide during the high temperature carbonization process. In subsequent acid dissolution and electrochemical exfoliation process, these unstable Fe and Mo compounds would dissolve (Figure S10, ESI), creating adequate defects and vacancies to trap other metal elements.

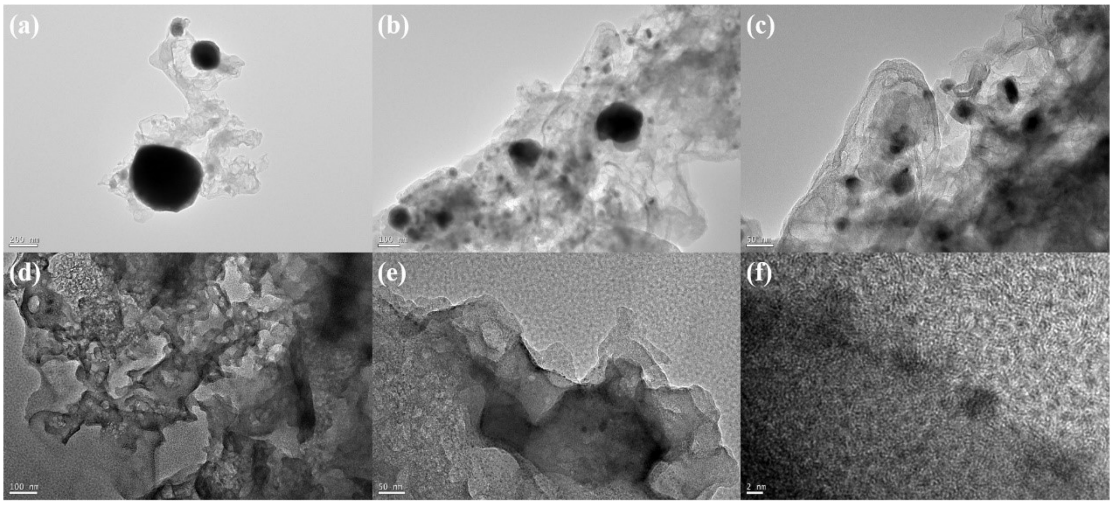

Figure S10. TEM images of FeMo@C before (a,b,c) and after (d,e,f) acid dissolution and electrochemical exfoliation process.

Notes for Figure S10. After the electrochemical modification process, some powder was taken off the nickel foam and characterized by TEM. It can be seen clearly that these unstable Fe and Mo compounds in FeMo@C before modification would dissolve. Therefore, all Mo and part of Fe were dissolved by acid and vacancies were created among the FeMo@C, which could trap Ni, Cu and Pt atoms subsequently.

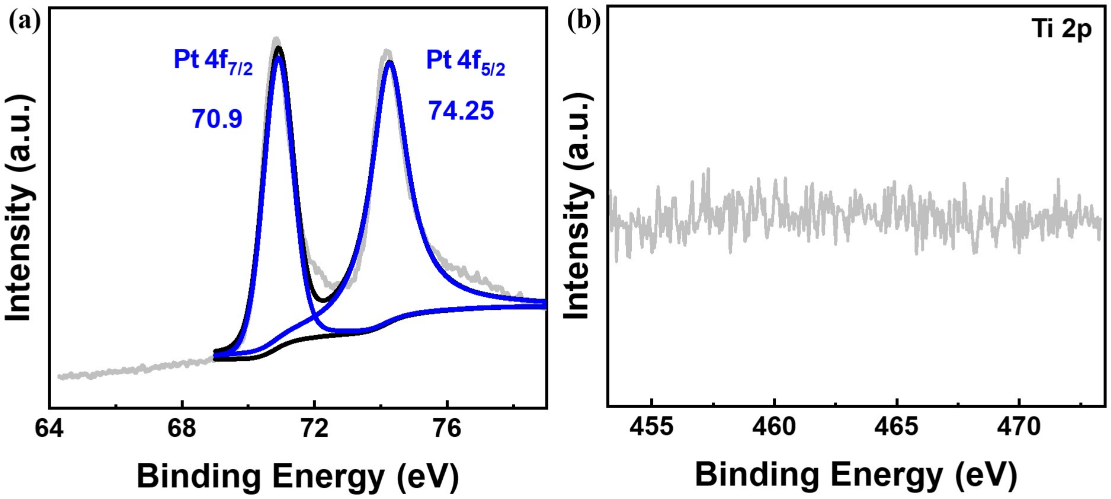

Figure S11. (a) High-resolution XPS Pt 4f spectrum of PtC; (b) High-resolution XPS Ti 2p spectrum of NFFeCuPt.

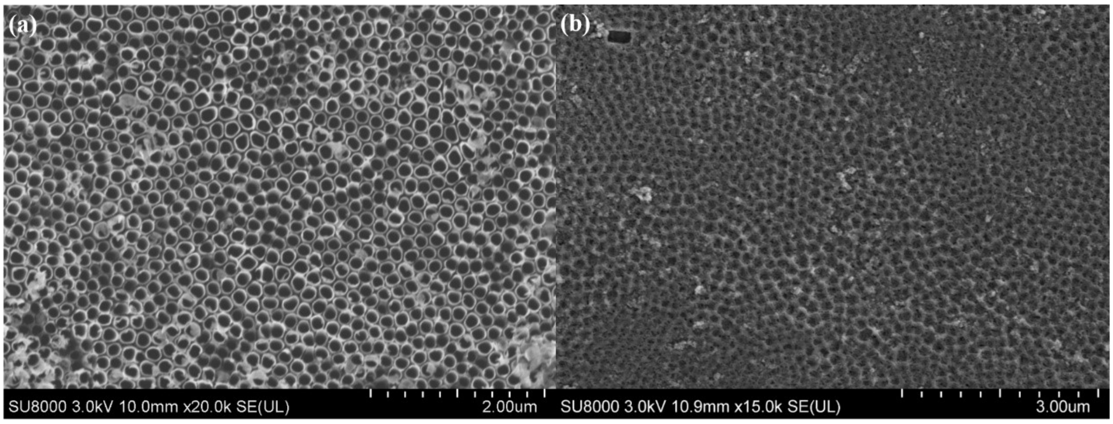

Figure S12. SEM images of TiO_2 NTs before (a) and after being used as CE for manufacturing 100 sheets of NFFeCuPt.

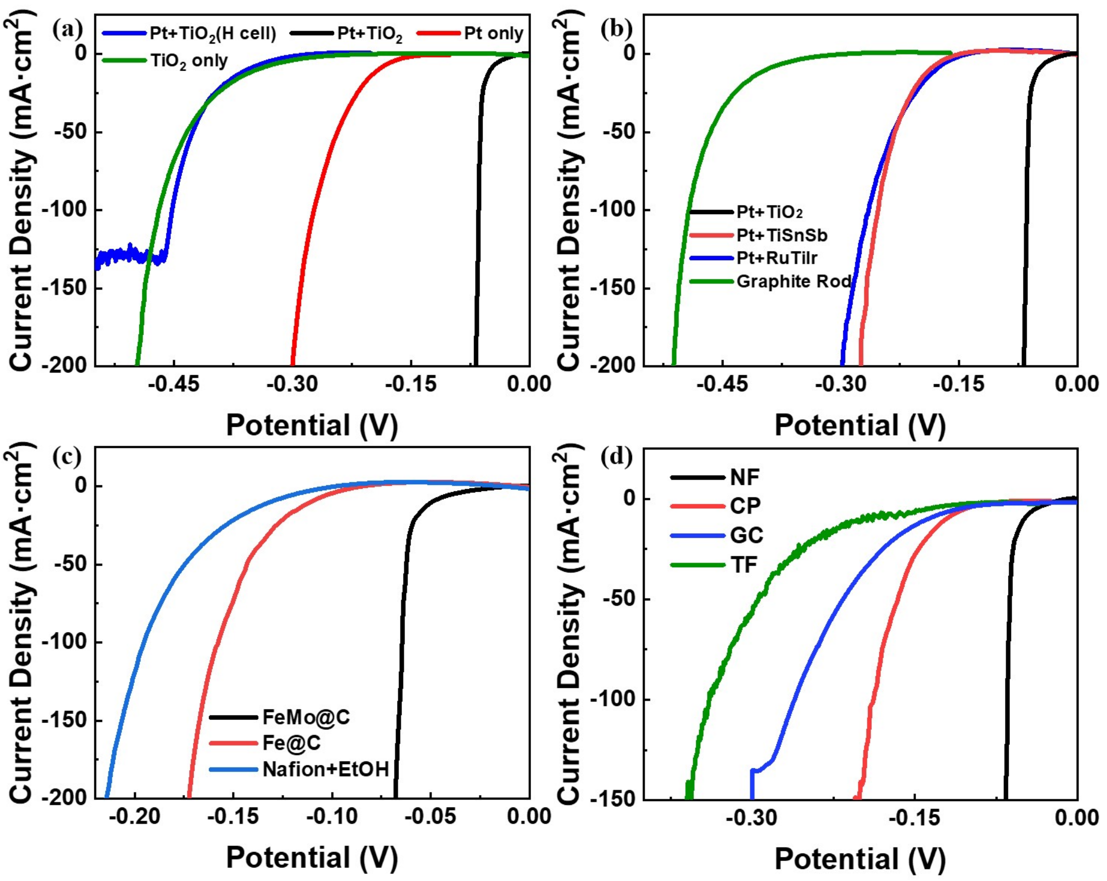

Figure S13. LSV polarization curves of NFFeCuPt after electrochemical modifications except changing TiO_2 NTs+platinum electrode holder into different CEs (a,b); changing FeMo@C into different loading materials (c) and changing NF into different WEs for loading FeMo@C (d).

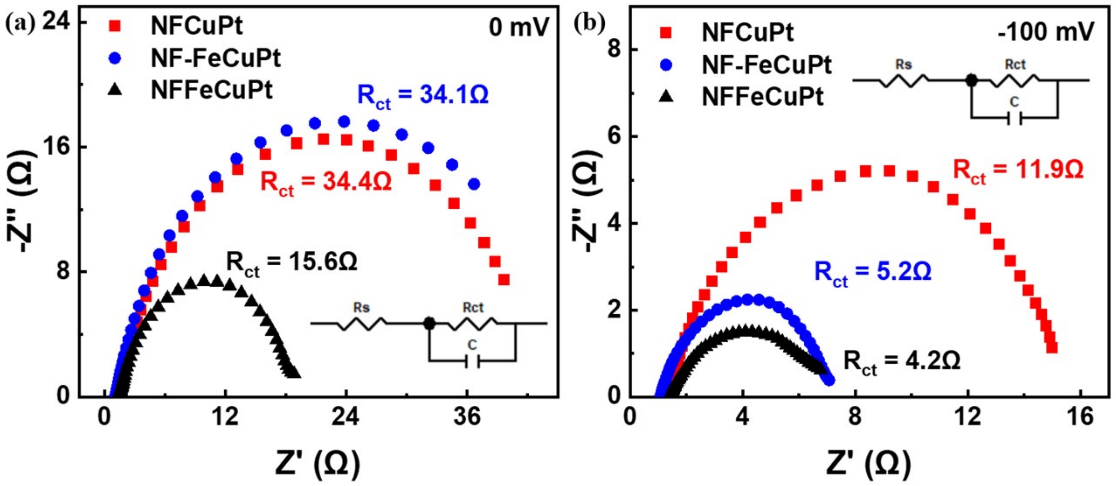

Figure S14. Electrochemical impedance spectra of NFCuPt, NF-FeCuPt and NFFeCuPt at overpotential 0 (a) and -100 mV (b) in 0.5 M H_2SO_4.

Notes for Figure S14. NFFeCu-Pt was obtained under the same conditions as NFFeCuPt for comparison except for changing the TiO_2 NTs CE into a Pt foil. NFCuPt and NF-FeCuPt were obtained under the same conditions as NFFeCuPt for comparison except for adding no FeMo@C or changing the FeMo@C into Fe@C.

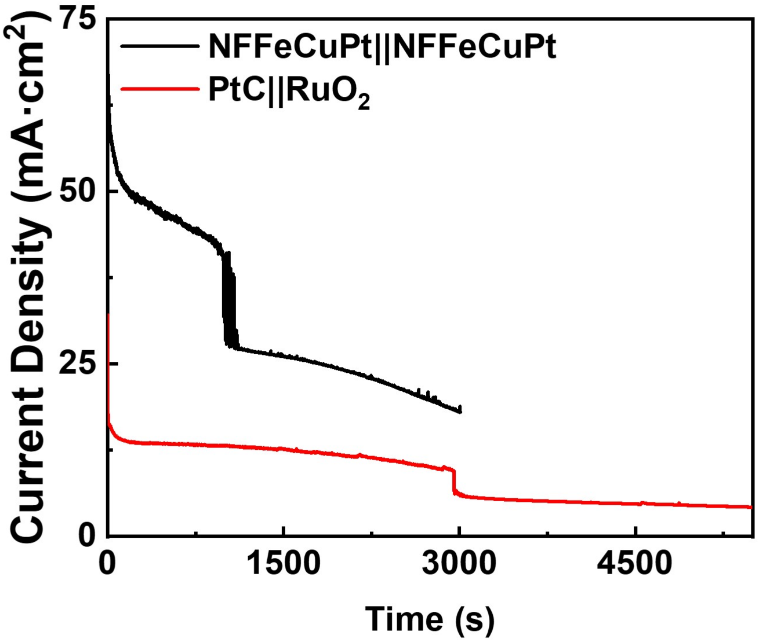

Figure S15. Potentiostatic tests of electrode pairs including NFFeCuPt||NFFeCuPt and PtC||RuO in 0.5 M H_2SO_4. The anodic NF dissolved and fractured obviously after electrolysis for less than 3000s.

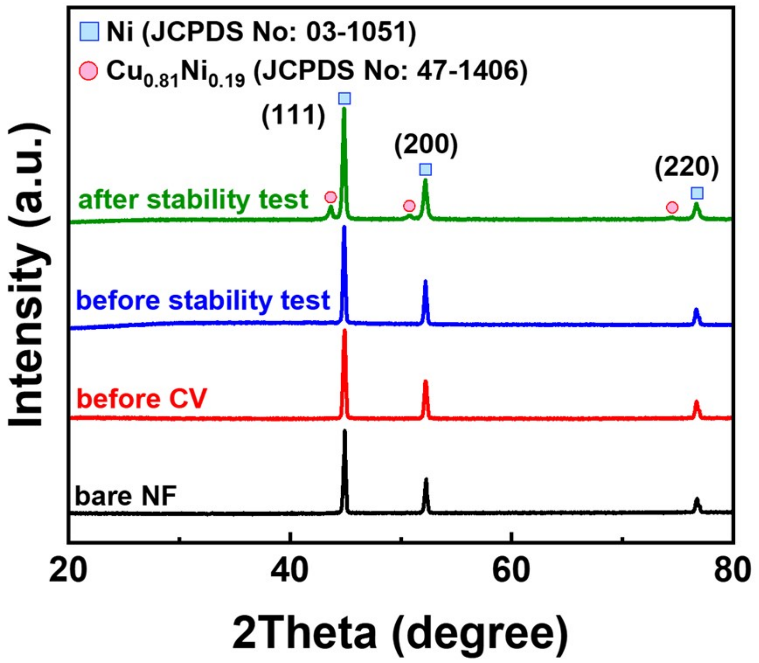

Figure S16. XRD patterns of bare NF, NFFeCuPt (before CV modification), NFFeCuPt (after CV modification and before stability test in Electrolyte 1) and NFFeCuPt (after stability test in Electrolyte 1).

Notes for Figure S16. It is evident that before and after loading FeMo@C, only Ni peaks of the Ni foam electrode can be detected. The diffraction peaks at 44.8°, 52.2°, and 76.8° correspond to the indexed planes of Ni (111), (200) and (220), respectively (JCPDS No: 03-1051). This is most likely because the amount of FeMo@C in the load is too small (only 0.6 mg), so the signal is shielded by a strong Ni peak. However, after CV modification and before the stability test, no Cu crystalline phase was detected, which is probably because the introduced Cu during CV modification stage mainly exists in the form of Cu^{+} and Cu^{2+} embedded among the carbon matrix rather than the crystalline alloy phase or elemental copper phase. This is consistent with the conclusion of semi-in-situ XPS (Fig. 2d). It is worth noting that after the stability test in Electrolyte 1, a new phase ($Cu_{0.81}Ni_{0.19}$) can be detected in XRD detection, which can be attributed to the formation of a new protective layer of Cu in the form of alloy on the surface of NF. The diffraction peaks at 43.6°, 50.8°, and 74.6° correspond to the indexed planes of $Cu_{0.81}Ni_{0.19}$ (111), (200) and (220), respectively (JCPDS No: 47-1406). The fact that this new phase is detected by XRD after the stability test in the Electrolyte 1 indicates that the CuNi protective layer is formed as a bulk phase on the entire surface of the NF electrode and demonstrates that part of the Cu^{2+} ions in the Electrolyte 1 are reduced to elemental form on the surface of the NF electrode.

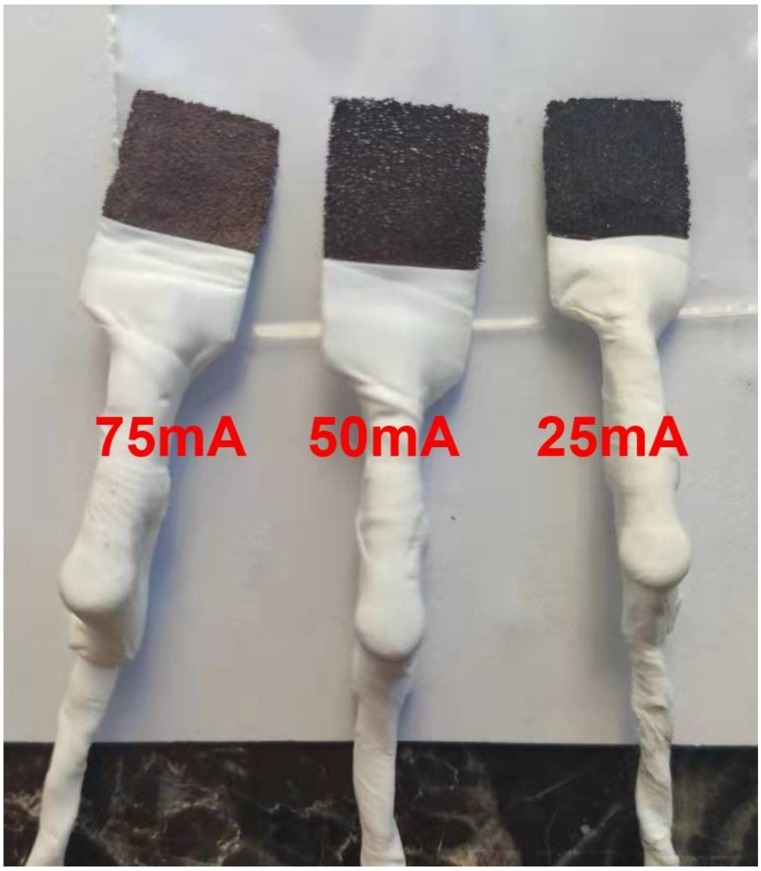

Figure S17. Optical pictures of NFFeCuPt after potentiostatic tests in different current densities in Electrolyte 1.

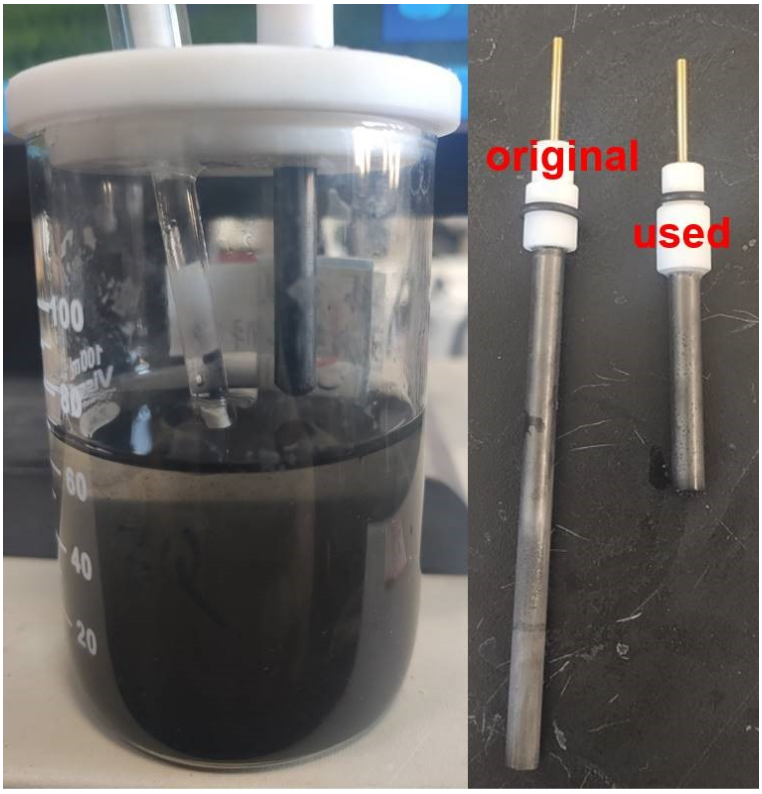

Figure S18. Optical pictures of graphite rod CE completely dissolved after stability test for about 300 h under acidic solution.

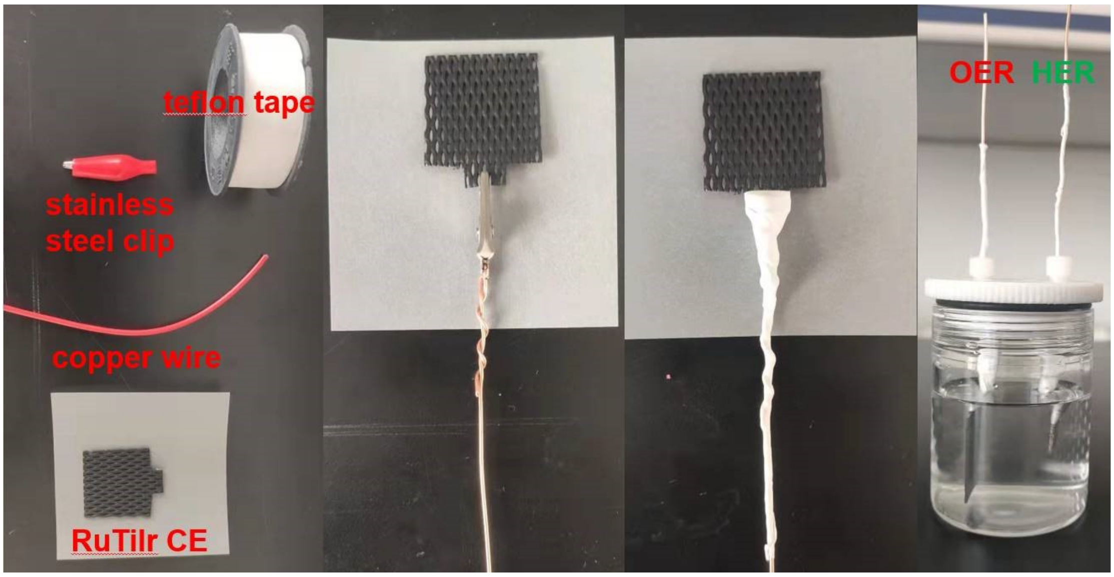

Figure S19. Schematic diagram of EWS cell assembled by WE (NFFeCuPt) and CE (RuTiIr) in acidic environment.

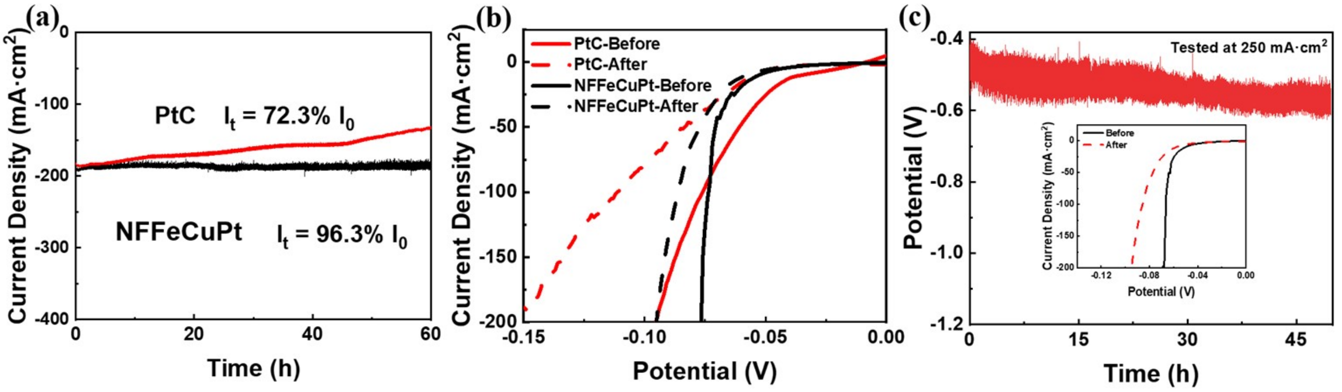

Figure S20. (a) Potentiostatic tests of NFFeCuPt and PtC at a current density about 200 mA cm^2; (b) LSV polarization curves of different WEs before and after potentiostatic tests; (c) chronopotentiometric test of NFFeCuPt at 250 mA cm^2 and inset is the LSV curves before and after chronopotentiometric test. All tests were carried out in Electrolyte 1.

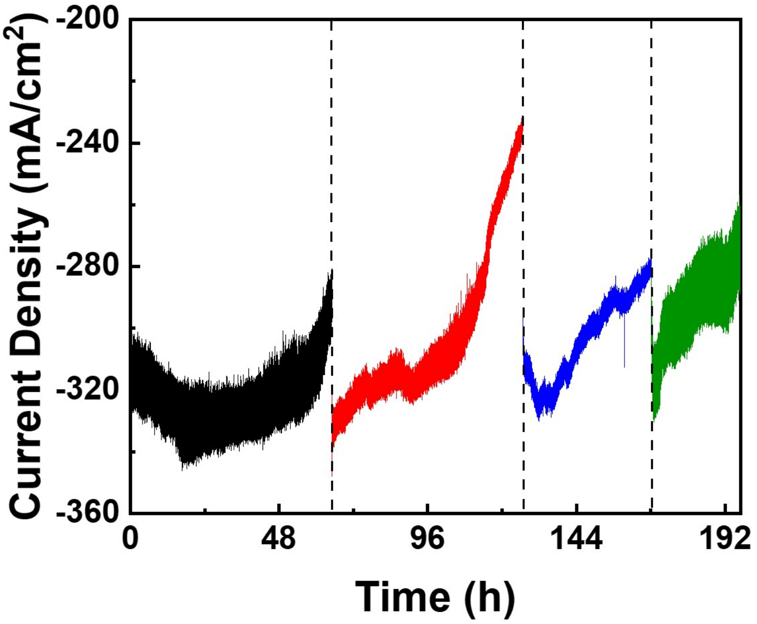

Figure S21. Potentiostatic tests of NFFeCuPt at current density about 300 mA cm^2 in the electrolyte 1. WE was treated with sulfuric acid and Electrolyte 1 was renewed after a period of time (the dotted line in the figure). Then potentiostatic tests were restarted and the current density restored to the initial value.

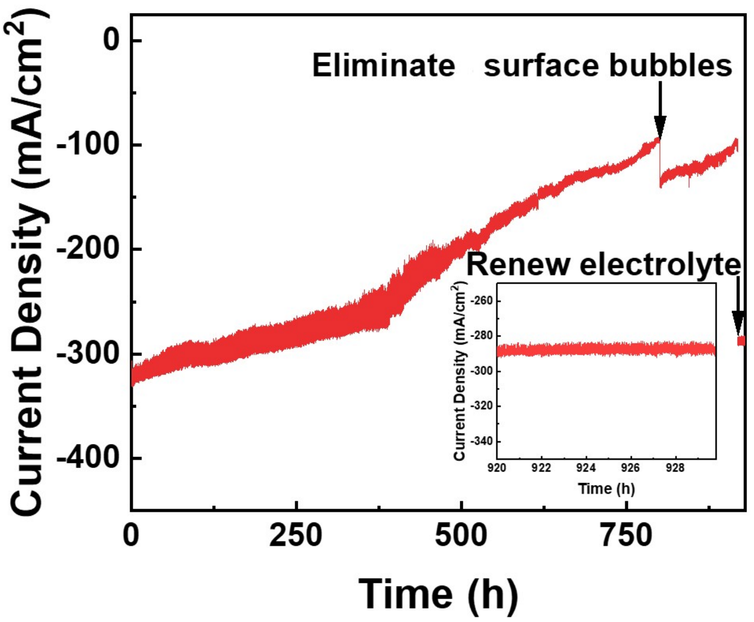

Figure S22. Potentiostatic test of NFFeCuPt at current density about 300 mA cm^2 in Electrolyte 1 with a larger electrolytic cell (500 mL) and inset is the enlarged figure of the final 10 h of the potentiostatic test after renewing the electrolyte and refreshing WE.

Notes for Figure S22. The attenuation rate of current density is greatly lower than that of small electrolytic cell because the concentration of Ni^{2+} in the larger cell increased more slowly than in the smaller one. Besides, HER performance was recovered after cleaning NFFeCuPt with 0.5 M H_2SO_4 and renewing Electrolyte 1.

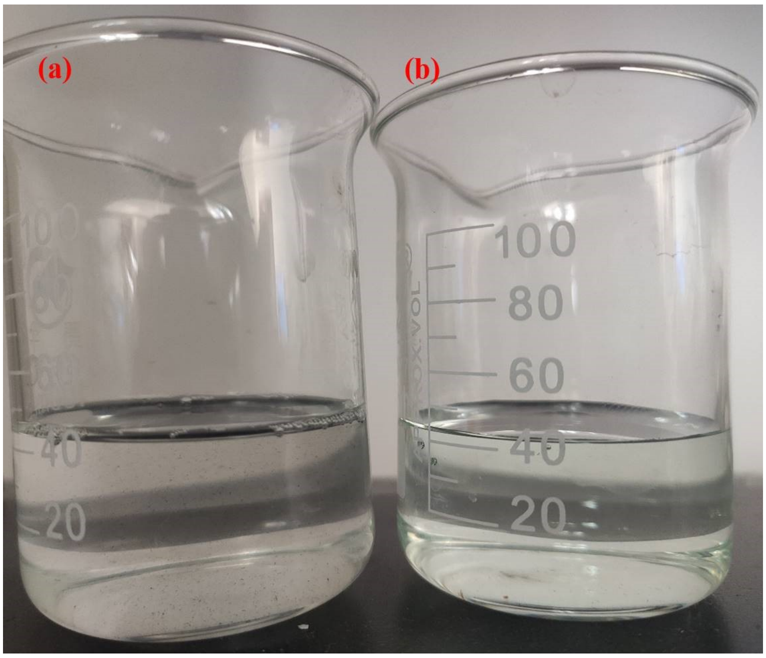

Figure S23. Electrolytes of NFFeCuPt after potentiostatic tests for 300 h at current densities of 25 mA cm^2 (a) and 300 mA cm^2 (b).

Notes for Figure S23. The dissolved Ni^{2+} ions, which were involved in the formation of the double electric layer, prevented the active sites in NFFeCuPt from accessing the reactants (H^+) due to the cathode potential. But it did not damage NFFeCuPt because the electrode performance could be restored once the electrolyte was renewed (Figure S20, Supporting Information). To further confirm the hypothesis, a larger electrolytic cell (500 mL, and original one is 100 mL) was used to repeat the potentiostatic test (Figure S22, Supporting Information).

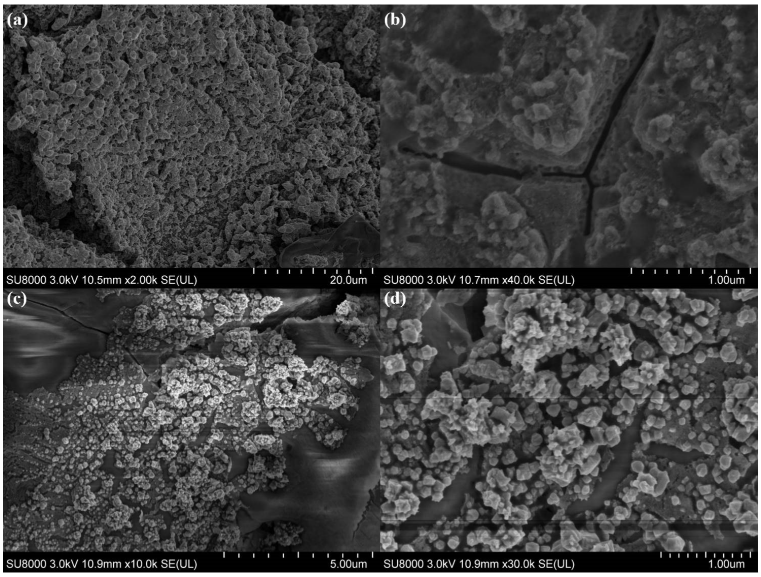

Figure S24. SEM images of NFFeCuPt (after potentiostatic test in Electrolyte 1) before (a,b) and after (c,d) H_2SO_4 treatment.

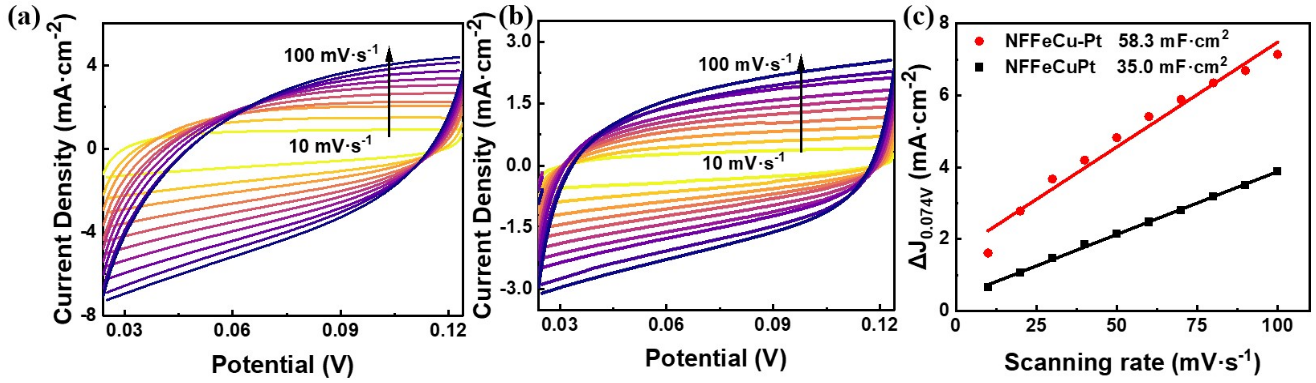

Figure S25. CV curves of NFFeCu-Pt (a) and NFFeCuPt (b) in different scanning rates; C_{dl} calculated with ΔJ at 0.074 (c); Electrolyte: 1 M KOH.

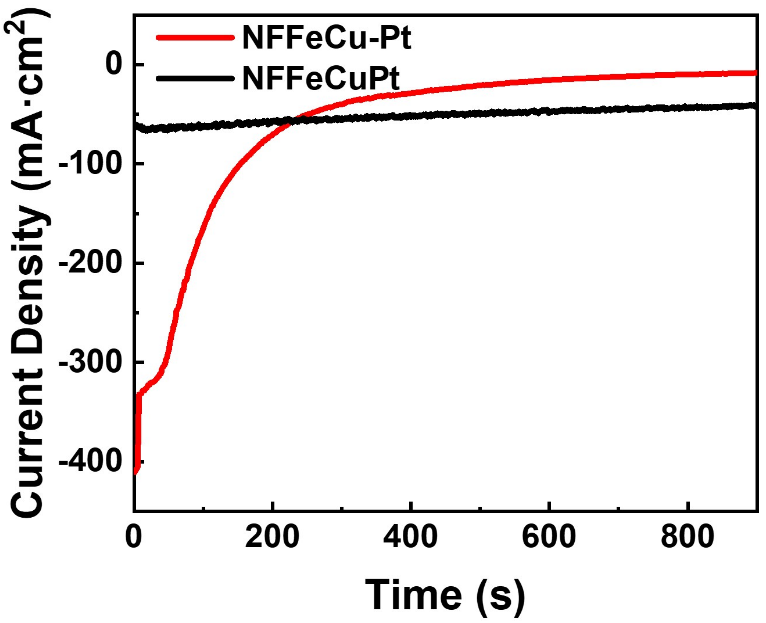

Figure S26. Potentiostatic plots of NFFeCuPt and NFFeCu-Pt in 0.5 M H_2SO_4.

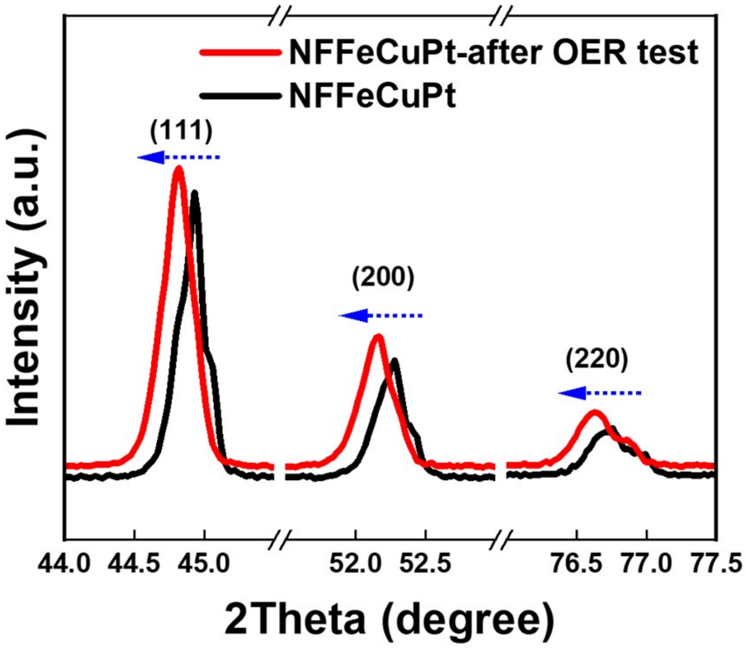

Figure S27. XRD patterns of NFFeCuPt before and after OER test in 1 M KOH.

Notes for Figure S27. The XRD peaks of Ni (111), (200) and (220) are obviously shifted after OER test. This is most likely because oxygen atoms are inserted into the original Ni lattice, causing the lattice expansion of Ni, resulting in a decrease of the 2θ angle[13]. Additionally, according to some previous reports[14, 15], the voltage required for oxidation of Ni is far lower than the conditions for OER (1.23V vs. RHE.) to occur. Thus it is reasonable to assume that oxygen atoms are inserted into the lattice of the nickel under this oxygen-rich alkaline condition.

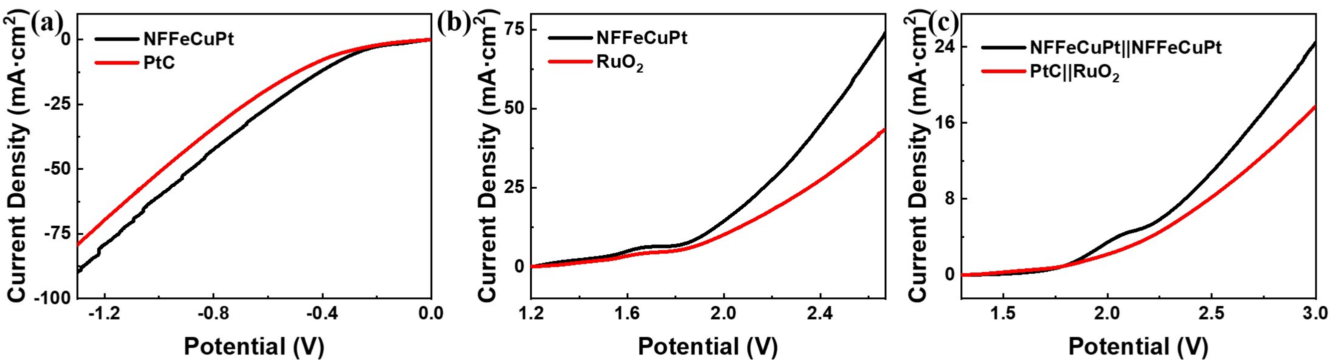

Figure S28. Electrochemical performance of NFFeCuPt for HER (a), OER (b) and EWS (c) in neutral 0.1 M PBS.

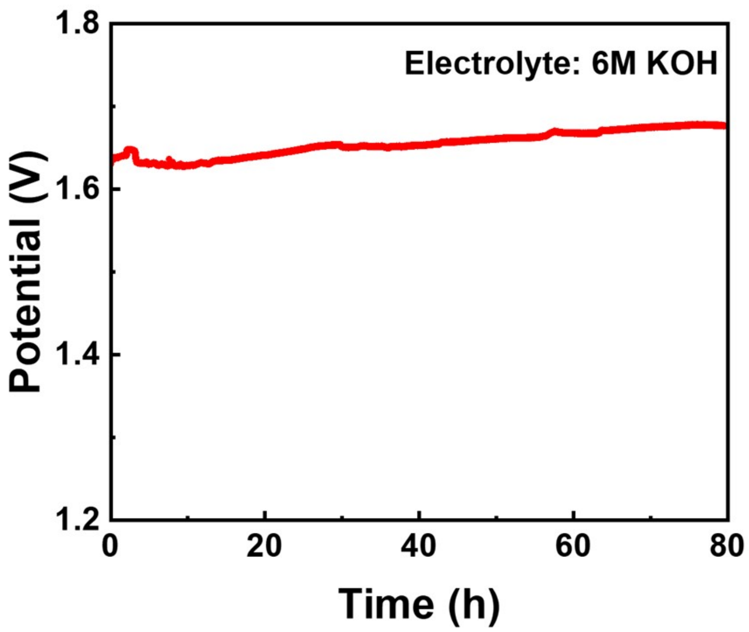

Figure S29. Chronopotentiometric test of NFFeCuPt at 100 mA·cm^2 in 6 M KOH.

Notes for Figure S29. The chronopotentiometric test was carried out using NFFeCuPt as WE (anode), graphite rods as CE (cathode) and Hg/HgO (1 M KOH electrolyte) electrode as RE. The electrolyte was 6 M KOH. The result exhibits that NFFeCuPt have excellent stability compared with some similar work (**Table S5**) even under highly alkaline environments.

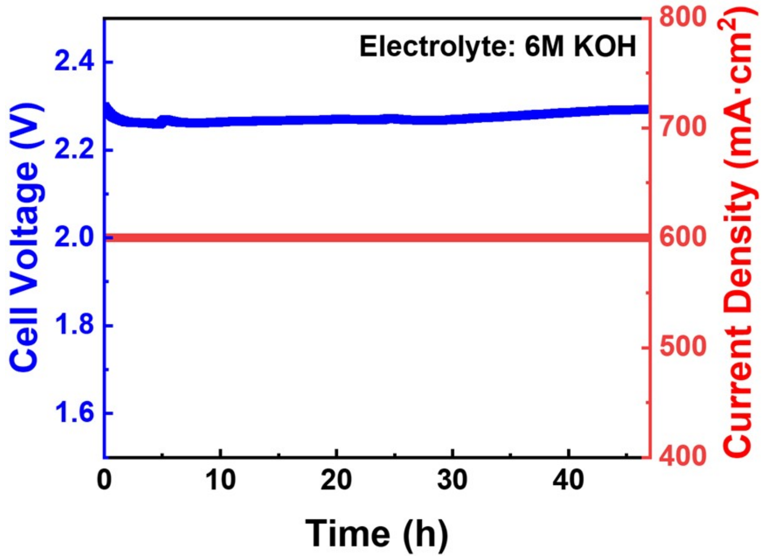

Figure S30. Stability test of NFFeCuPt under simulating working conditions (2.3V, 600 mA·cm^2) in 6 M KOH.

Notes for Figure S30. The stability test under simulating working conditions was carried out using the charge-discharge instrument (Neware CT-4008T-5V6A-S1) while NFFeCuPt as WE (anode) and RuTiIr DSA electrode as CE (cathode). The electrolyte was 6 M KOH. The parameters of the charge-discharge instrument are set as constant voltage charging mode(voltage=2.3V without **iR compensation**) with cut-off current at 600 mA.

Table S1. Comparison of HER performance in acidic electrolyte.

Electrocatalysts	Overpotential(j_{10})	Overpotential(j_{100})	Ref
BPIr_be	25	N/A (~80 mV @ j = 200 mA cm^{-2})	[16]
RuMn NSBs	18	N/A	[17]
Pt/Co	7	54	[18]
PtNC/S–C	11	N/A	[19]
PtSA-NT-NF	30	88	[20]
$Mo_2TiC_2T_x$–Pt_{SA}	30	73	[21]
Pt SAs/DG	23	~73	[22]
Pt@MoS_2/NiS_2	34	~98	[23]
**Commercial Pt/C**	**33**	**66**	**This work**
**NFFeCuPt**	**40**	**66**	**This work**

Table S2. Comparison of OER performance in acidic electrolyte.

Electrocatalysts	Overpotential(j_{10})	Overpotential(j_{100})	Ref
BPIr_be	290	400	[16]
BPIr_sur	~290	430	[16]
Ir	~340	500	[16]
RuMn NSBs	196	N/A	[17]
Ir-C≡	256	N/A	[24]
$IrO_x/SrIrO_3$	270	N/A	[25]
Ir-MnO2	218	283	[26]
Cu-doped RuO2	188	N/A	[27]
**RuO_2**	**218**	**367**	**This work**
**NFFeCuPt**	**159**	**273**	**This work**

Table S3. Comparison of HER performance in alkaline electrolyte.

Electrocatalysts	Overpotential(j_{10})	Overpotential(j_{100})	Ref
PtRu/BP	22	N/A	[28]
Pt/BP	64	N/A	[28]
BPIr_be	2	59	[16]
BPIr_sur	105	~250	[16]
RuMn NSBs	20	N/A	[17]
Ni(Cu)VO_x	10	42	[29]
Pt_{SA}-NiO/Ni	26	85	[30]
NiFe@Pt	70	N/A	[31]
**Commercial Pt/C**	**25**	**102**	**This work**
**NFFeCuPt**	**39**	**97**	**This work**

Table S4. Comparison of OER performance in alkaline electrolyte.

Electrocatalysts	Overpotential(j_{10})	Overpotential(j_{100})	Ref
BPIr_be	290	380	[16]
Ir	~290	390	[16]
RuMn NSBs	175	N/A	[17]
Ir-C≡	300	N/A	[24]
RuCu NSs	234	N/A	[32]
a-$RuTe_2$ PNRs	285	N/A	[33]
RuIrOx	250	N/A	[34]
0.27-RuO_2@C	~200	N/A (~300 mV @ j = 50 mA cm^{-2})	[35]
**RuO_2**	**310**	**440**	**This work**
**NFFeCuPt**	**230**	**330**	**This work**

Table S5. Comparison of OER stability in alkaline electrolyte.

Electrocatalysts	Stablizing time (h)	Electrolyte	Current density (mA·cm^2)	Ref
CoNi@NCNTs	4.5	0.1 M KOH	10	[36]
NPCs	30	6 M KOH	10	[37]
SSC	100	6 M KOH	50	[38]
Co-Ni-S@NSPC	60	0.1 M KOH	10	[39]
BSCF	150	6 M KOH	<10	[40]
CoFeP	30	1 M KOH	10	[41]
α−MnO_2-C	50	6 M KOH	10	[42]
Co_3O_4/MnO_2/PQ-7	54	6 M KOH	10	[43]
$Fe_{0.1}Ni_{0.9}Co_2O_4$	65	6 M KOH	10	[44]
**NFFeCuPt**	**80**	**6 M KOH**	**100**	**This work**
**NFFeCuPt**	**47**	**6 M KOH**	**600**	**This work**

References

1. L. H. Ai, C. H. Zhang, L. L. Li and J. Jiang, *Applied Catalysis B-Environmental*, 2014, **148**, 191-200.
2. Z. Q. Zhang, T. Wang, S. Q. Zhang, K. D. Yao, Y. Q. Sun, Y. Liu, X. Z. Wang and W. M. Huang, *Applied Surface Science*, 2021, **551**, 149425.
3. Z. Q. Zhang, Y. Zhang, K. D. Yao, W. M. Huang and T. J. Wang, *Inorganica Chimica Acta*, 2021, **528**, 120599.
4. Z. Q. Zhang, L. C. Cong, Z. C. Yu, L. Qu, M. M. Qian and W. M. Huang, *Materials Advances*, 2020, **1**, 54-60.
5. Z. Zhang, L. Cong, Z. Yu, L. Qu and W. Huang, *Materials Today Energy*, 2020, **16**, 100387.
6. J. Duan, S. Chen and C. Zhao, *Nat Commun*, 2017, **8**, 15341.
7. L. Zhuang, Y. Jia, H. Liu, Z. Li, M. Li, L. Zhang, X. Wang, D. Yang, Z. Zhu and X. Yao, *Angew Chem Int Ed Engl*, 2020, **59**, 14664-14670.
8. X. H. Li, W. L. Guo, Z. H. Liu, R. Q. Wang and H. Liu, *Applied Surface Science*, 2016, **369**, 130-136.
9. R. S. Mann and K. C. Khulbe, *Bulletin of the Chemical Society of Japan*, 1975, **48**, 1021-1023.
10. Z. C. Li, J. J. Ma, B. Zhang, C. X. Song and D. B. Wang, *Crystengcomm*, 2017, **19**, 1479-1485.
11. L. Jiao, R. Zhang, G. Wan, W. Yang, X. Wan, H. Zhou, J. Shui, S. H. Yu and H. L. Jiang, *Nat Commun*, 2020, **11**, 2831.
12. Z. Q. Zhang, K. D. Yao, L. C. Cong, Z. C. Yu, L. N. Qu and W. M. Huang, *Catalysis Science & Technology*, 2020, **10**, 1336-1342.
13. L. Zhao, Y. Zhang, Z. Zhao, Q. H. Zhang, L. B. Huang, L. Gu, G. Lu, J. S. Hu and L. J. Wan, *Natl Sci Rev*, 2020, **7**, 27-36.
14. L. Wang, *Micro & Nano Letters*, 2017, **12**, 166-169.
15. V. S. Kumbhar, M. H. Cho, J. Lee, W. K. Kim, M. Lee, Y. R. Lee and J.-J. Shim, *New Journal of Chemistry*, 2017, **41**, 10584-10591.
16. J. Mei, T. He, J. Bai, D. Qi, A. Du, T. Liao, G. A. Ayoko, Y. Yamauchi, L. Sun and Z. Sun, *Adv Mater*, 2021, **n/a**, e2104638.
17. L. Li, L. Bu, B. Huang, P. Wang, C. Shen, S. Bai, T. S. Chan, Q. Shao, Z. Hu and X. Huang, *Adv Mater*, 2021, **33**, e2105308.
18. L. H. Sun, Q. Y. Li, S. N. Zhang, D. Xu, Z. H. Xue, H. Su, X. Lin, G. Y. Zhai, P. Gao, S. I. Hirano, J. S. Chen and X. H. Li, *Angew Chem Int Ed Engl*, 2021, **60**, 25766-25770.
19. Q. Q. Yan, D. X. Wu, S. Q. Chu, Z. Q. Chen, Y. Lin, M. X. Chen, J. Zhang, X. J. Wu and H. W. Liang, *Nat Commun*, 2019, **10**, 4977.
20. L. Zhang, L. Han, H. Liu, X. Liu and J. Luo, *Angew Chem Int Ed Engl*, 2017, **56**, 13694-13698.
21. J. Q. Zhang, Y. F. Zhao, X. Guo, C. Chen, C. L. Dong, R. S. Liu, C. P. Han, Y. D. Li, Y. Gogotsi and G. X. Wang, *Nature Catalysis*, 2018, **1**, 985-992.
22. Y. Qu, B. Chen, Z. Li, X. Duan, L. Wang, Y. Lin, T. Yuan, F. Zhou, Y. Hu, Z. Yang, C. Zhao, J. Wang, C. Zhao, Y. Hu, G. Wu, Q. Zhang, Q. Xu, B. Liu, P. Gao, R. You, W. Huang, L. Zheng, L. Gu, Y. Wu and Y. Li, *J Am Chem Soc*, 2019, **141**, 4505-4509.
23. Y. Guan, Y. Feng, J. Wan, X. Yang, L. Fang, X. Gu, R. Liu, Z. Huang, J. Li, J. Luo, C. Li and Y. Wang, *Small*, 2018, **14**, e1800697.
24. Y. Peng, Q. M. Liu, B. Z. Lu, T. He, F. Nichols, X. Hu, T. Huang, G. Huang, L. Guzman, Y. Ping and S. W. Chen, *Acs Catalysis*, 2021, **11**, 1179-1188.
25. L. C. Seitz, C. F. Dickens, K. Nishio, Y. Hikita, J. Montoya, A. Doyle, C. Kirk, A. Vojvodic, H. Y. Hwang, J. K. Norskov and T. F. Jaramillo, *Science*, 2016, **353**, 1011-1014.
26. Z. P. Shi, Y. Wang, J. Li, X. Wang, Y. B. Wang, Y. Li, W. L. Xu, Z. Jiang, C. P. Liu, W. Xing and J. J. Ge, *Joule*, 2021, **5**, 2164-2176.
27. J. Su, R. Ge, K. Jiang, Y. Dong, F. Hao, Z. Tian, G. Chen and L. Chen, *Adv Mater*, 2018, **30**, e1801351.
28. W. H. Li, M. S. Li, J. J. Li, J. N. Liang, K. R. Adair, Y. F. Hu, Q. F. Xiao, X. Y. Cui, R. Y. Li, F. Brandys, R. Divigalpitiya, T. K. Sham and X. L. Sun, *Acs Applied Nano Materials*, 2020, **3**, 7508-7515.
29. Y. Li, X. Tan, R. K. Hocking, X. Bo, H. Ren, B. Johannessen, S. C. Smith and C. Zhao, *Nat Commun*, 2020, **11**, 2720.
30. K. L. Zhou, Z. Wang, C. B. Han, X. Ke, C. Wang, Y. Jin, Q. Zhang, J. Liu, H. Wang and H. Yan, *Nat Commun*, 2021, **12**, 3783.
31. S. Xue, R. W. Haid, R. M. Kluge, X. Ding, B. Garlyyev, J. Fichtner, S. Watzele, S. Hou and A. S. Bandarenka, *Angew Chem Int Ed Engl*, 2020, **59**, 10934-10938.
32. Q. Yao, B. Huang, N. Zhang, M. Sun, Q. Shao and X. Huang, *Angew Chem Int Ed Engl*, 2019, **58**, 13983-13988.
33. J. Wang, L. Han, B. Huang, Q. Shao, H. L. Xin and X. Huang, *Nat Commun*, 2019, **10**, 5692.
34. Z. Zhuang, Y. Wang, C. Q. Xu, S. Liu, C. Chen, Q. Peng, Z. Zhuang, H. Xiao, Y. Pan, S. Lu, R. Yu, W. C. Cheong, X. Cao, K. Wu, K. Sun, Y. Wang, D. Wang, J. Li and Y. Li, *Nat Commun*, 2019, **10**, 4875.
35. H. S. Park, J. Yang, M. K. Cho, Y. Lee, S. Cho, S. D. Yim, B. S. Kim, J. H. Jang and H. K. Song, *Nano Energy*, 2019, **55**, 49-58.
36. Y. Xie, C. Feng, Y. Guo, S. Li, C. Guo, Y. Zhang and J. Wang, *Applied Surface Science*, 2021, **536**, 147786.
37. B. Guo, R. Ma, Z. Li, S. Guo, J. Luo, M. Yang, Q. Liu, T. Thomas and J. Wang, *Nano-Micro Letters*, 2020, **12**, 20.
38. X. Zhang, K. Tsay, W. Qu and J. Fahlman, *Journal of Energy Storage*, 2018, **20**, 520-528.
39. W. Fang, H. Hu, T. Jiang, G. Li and M. Wu, *Carbon*, 2019, **146**, 476-485.
40. J.-I. Jung, M. Risch, S. Park, M. G. Kim, G. Nam, H.-Y. Jeong, Y. Shao-Horn and J. Cho, *Energy & Environmental Science*, 2016, **9**, 176-183.
41. D.-S. Pan, P. Chen, L.-L. Zhou, J.-H. Liu, Z.-H. Guo and J.-L. Song, *Journal of Power Sources*, 2021, **498**, 229859.
42. P.-C. Li, C.-C. Hu, T.-H. You and P.-Y. Chen, *Carbon*, 2017, **111**, 813-821.
43. X. Li, F. Dong, N. Xu, T. Zhang, K. Li and J. Qiao, *ACS Applied Materials & Interfaces*, 2018, **10**, 15591-15601.
44. Y.-T. Lu, Y.-J. Chien, C.-F. Liu, T.-H. You and C.-C. Hu, *Journal of Materials Chemistry A*, 2017, **5**, 21016-21026.
